# Catalytic application of sulfamic acid-functionalized magnetic Fe_3_O_4_ nanoparticles (SA-MNPs) for protection of aromatic carbonyl compounds and alcohols: experimental and theoretical studies†

**DOI:** 10.1039/d0ra09087e

**Published:** 2020-12-21

**Authors:** Sepideh Khaef, Mohammad Ali Zolfigol, Avat Arman Taherpour, Meysam Yarie

**Affiliations:** Department of Organic Chemistry, Faculty of Chemistry, Bu-Ali Sina University Hamedan Iran zolfi@basu.ac.ir mzolfigol@yahoo.com; Department of Organic Chemistry, Faculty of Chemistry, Razi University Kermanshah Iran avat.taherpour@gmail.com

## Abstract

Protection techniques of functional groups within the structure of organic compounds are important synthetic methods against unwanted attacks from various reagents during synthetic sequences. Acetal and thioacetal groups are well known as protective functional groups in organic reactions. In this study, acetalization of carbonyl compounds with diols and dithiols and methoxymethylation of alcohols with formaldehyde dimethyl acetal (FDMA) have been carried out using sulfamic acid-functionalized magnetic Fe_3_O_4_ nanoparticles (SA-MNPs) as a heterogeneous solid acid catalyst. Products were characterized by FT-IR and NMR spectroscopies. The structural and electronic properties of some products were computed by quantum mechanical (QM) methods. Depending on the stereochemistry and electronic properties that were obtained by computational results, we have suggested that hyperconjugation plays a key role in the structural properties of 2-phenyl-1,3-dioxolane derivatives, and also the electron transfer between π-electrons of the aromatic ring with the 3d orbital of S-atoms influences the 2-phenyl-1,3-dithiane derivatives' structure.

## Abstract 2

Protection techniques of functional groups within the structure of organic compounds are important synthetic methods against unwanted attacks from various reagents during synthetic sequences.

## Graphical abstract

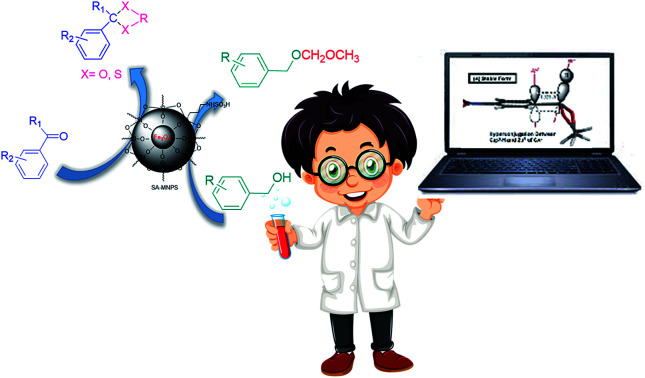

## Introduction

**Scheme 1:**
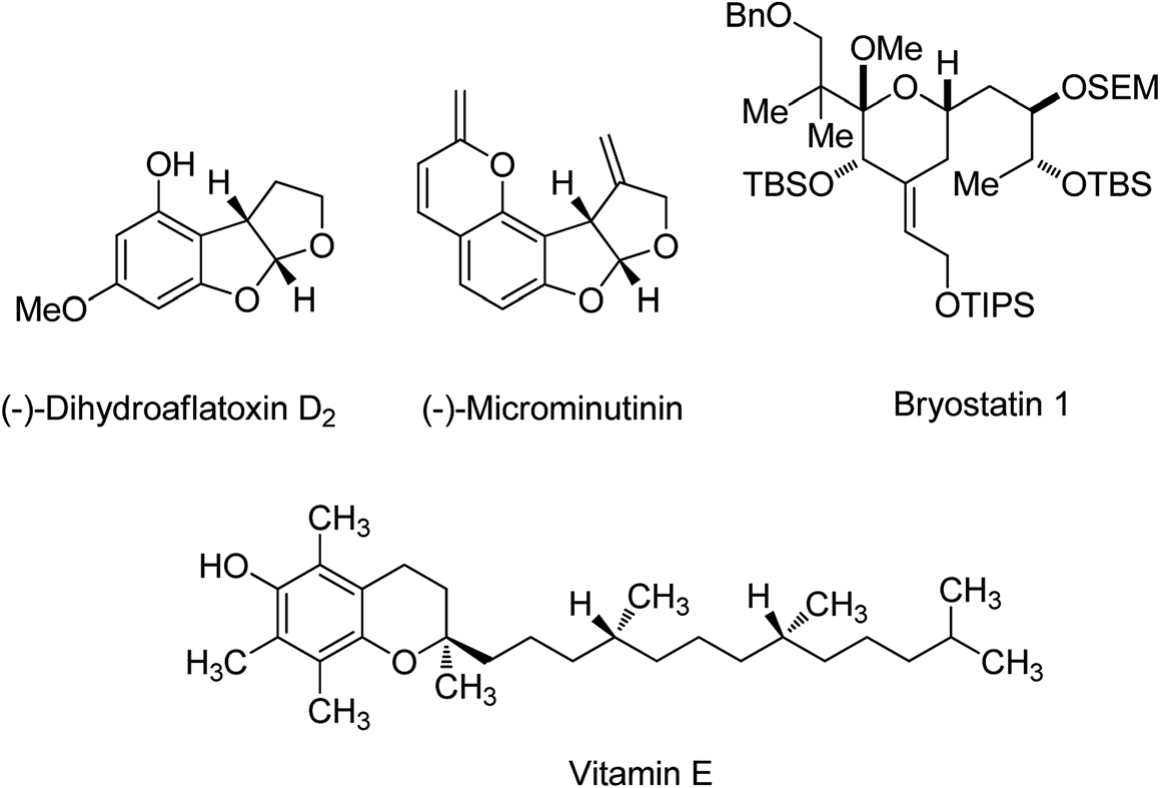
Samples of compounds where protection strategies take place in their synthesis.

Protection strategies of carbonyl compounds are well known as one of the most important branches of organic chemistry in the field of multistep total synthesis of enantiomerically pure natural and non-natural compounds.^1^ For example, protection strategies were applied in the synthetic processes of (−)-dihydroaflatoxin D_2_,^2^ (−)- & (+)-microminutinin,^2^ optically active vitamin E^3^ and bryostatin 1 (ref. 4) (Scheme 1).

The known protecting groups of carbonyl compounds are acetals, ketals, dithianes and acylals. One of the most useful protective groups in synthetic reactions are acetals.^5^ Due to the relatively stable toward a variety of reagents, these intermediates are noticeable and also, cyclic acetals are generally much easier to construct than open-chain acetals.^6^ Acetalization performed by reaction between an aldehyde with two equivalents (or an excess amount) of an alcohol, or a diol and elimination of water in acidic conditions. Likewise, using ketone derivatives instead of aldehydes was called ketalization in this kind of reaction.^5–7^

**Fig. 1:**
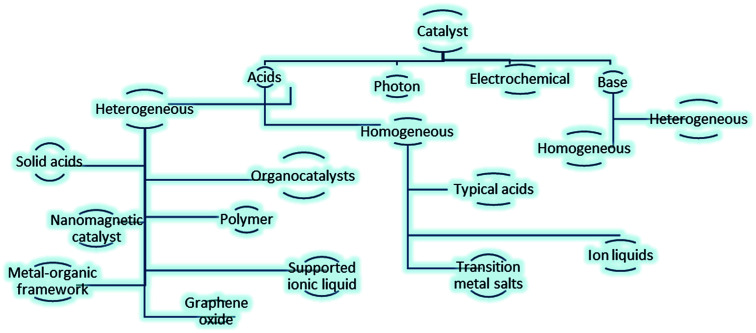
Varied catalytic systems applied for protection of carbonyls.^8–18^

Due to the great importance of these fundamental reactions, various catalysts have been designed and used for carbonyl protection. Fig. 1, portrayed varied catalytic systems including photons,^8^ electrochemical,^9^ bases^10^ and acids, like solid acids,^11^ organocatalysts,^12^ polymers,^13^ nanomagnetic catalyst,^14^ metal–organic framework,^15^ graphene oxide,^16^ transition metal salts^17^ and ion liquids^18^ in this field.

On the other hand, one of the most important purposes in new science is the application of easily separable catalysts which follows the green chemistry principles. Nano magnetic catalysts have been recently known as useful catalysts due to easy separation, reusability and fully dispersion in reaction media.^19^ The surface functionalization of magnetic nanoparticles is a well-designed method to convert homogeneous catalysts to heterogeneous ones.^20^ Acidic-functionalized magnetic nanoparticles are known as efficient acidic catalysts applied for a variety of organic reactions which need acidic conditions.^21^

**Scheme 2:**
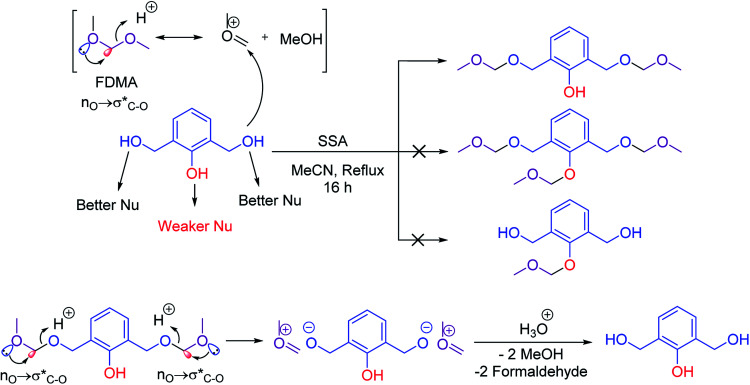
Chemoselective methoxymethylation of benzylic alcohols and its hydrolysis process.

Several methods have been reported for protection of alcohols with formaldehyde dimethoxy acetal (FDMA).^22^ The trace and impact of anomeric effect can be observed in methoxymethylation of alcohols. For example, the catalytic activity of silica sulfuric acid (SSA) was investigated for the chemoselective methoxymethylation of benzylic alcohols. It is suggested that the anomeric effect leads to activation of FDMA for protection of alcohols. In this case, the anomeric effect (n_O_ → 
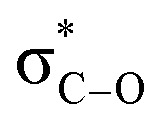
) leads to weakening of C–O bond and facilitates the protection process (Scheme 2).^22*a*^ It is worthy to mention that due to the resonance phenomenon, the phenolic hydroxy group is a weaker nucleophile than its alcoholic (benzylic hydroxy group) ones.

Commonly, preparation of methoxymethyl ethers from hydroxylic compounds has been carried out by formaldehyde dimethyl acetal (FDMA) used as a stable, low-cost, and accessible compound.^23^

**Fig. 2:**
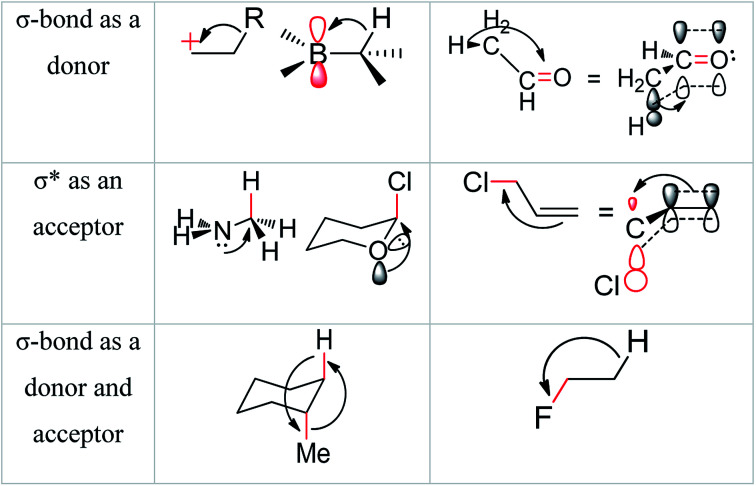
Examples of delocalizing interactions of hyperconjugation.^24*a*^

Hyperconjugation theorem enriches the concept of the molecular stabilizing effect which is occurred with interactions between electronic orbitals.^24^ In general, hyperconjugation was well defined as the interaction between electronic orbitals, for example by the use of conjugation between 2p and σ-bond molecular orbital. Few important examples of hyperconjugation's patterns were shown in Fig. 2.^24*a*^

**Fig. 3:**
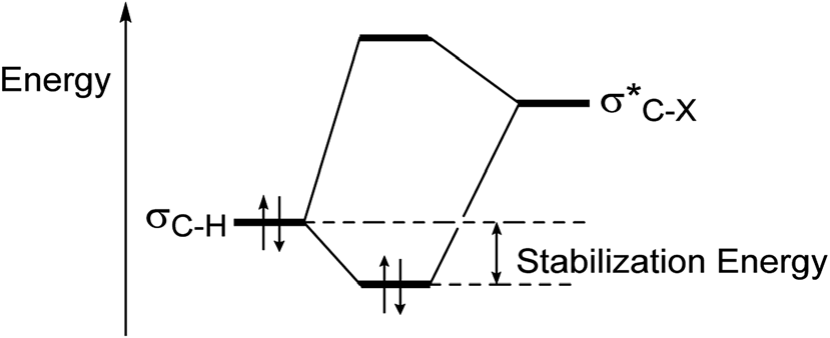
Energy lowering due to hyperconjugative interaction between σ_C–H_ and 
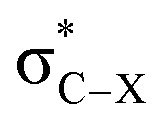
 orbitals.^24*a*^

Due to the interaction between two-electrons of the lower energy orbital (σ-bond or a lone pair) with the higher energy empty antibonding orbital, has made a stabilized molecule by hyperconjugation concept (Fig. 3).^24*a*^

**Scheme 3:**
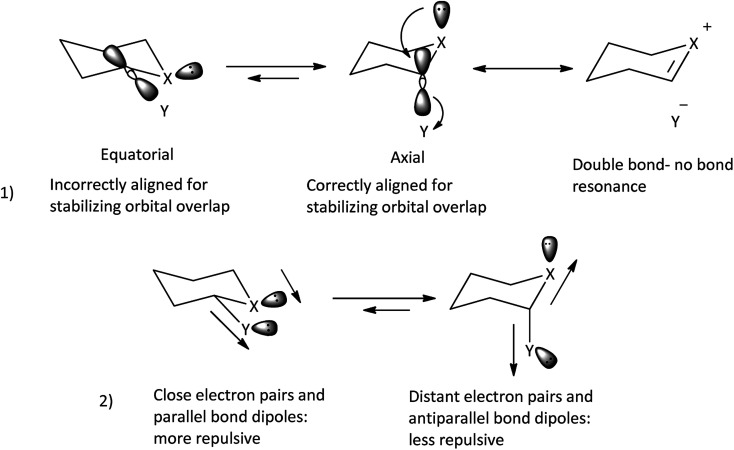
Factors make the anomeric effect.^25*a*^

Classical anomeric effect is one of the well-known stereoelectronic effects which is defined as the tendency of electronegative heteroatomic substituents positioned next to a heteroatom within a cyclohexane ring to prefer an axial rather than an equatorial position.^24*a*,25^ In the anomeric theory it is suggested that the existence of a stabilizing interaction between the lone pair of ring heteroatom and the anti-periplanar antibonding σ* orbital of the axial substituent bond at the anomeric carbon (n_X_ → 
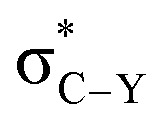
) leads to weakening of C–Y bond and increase negative charge on Y. Also, predicted dipole–dipole repulsion between the two electronegative atoms that one part of the ring and the other exocyclic, causes the preference for the smallest dipole moment with the axial conformation (Scheme 3).^25*a*^ Many experimental and computational evidence proved the stereoelectronic nature of the anomeric effect.^25^

## Computational methods and modeling details

**Scheme 4:**
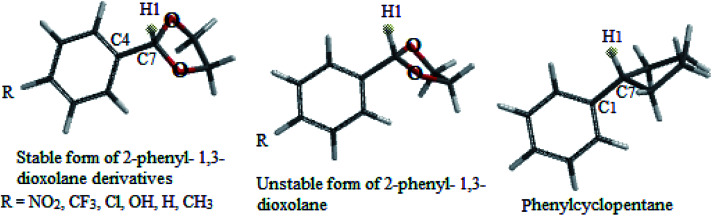
Stable and unstable forms of 2-phenyl-1,3-dioxolane derivatives and phenyl cyclopentane.

**Scheme 5:**
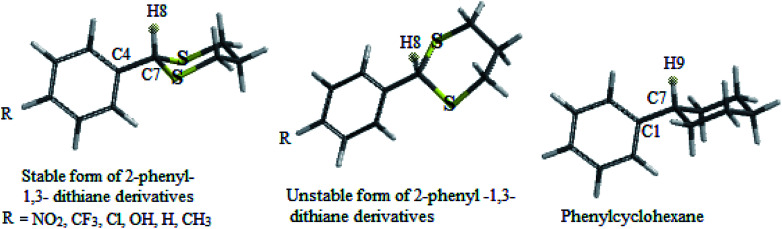
Stable and unstable forms of 2-phenyl-1,3-dithiane derivatives and phenyl cyclohexane.

In this study which is about the synthesis of acetals and thioacetals, some of the appropriate quantum mechanical (QM) methods were applied, in order to determine the most stable structure of the products. The DFT-B3LYP method was performed by Spartan'10 package.^26^ In this molecular modeling, the structures were minimized and optimized by B3LYP levels with the 6-31G* basis sets. The structural and electronic properties of some products (2-phenyl-1,3-dioxolane and 2-phenyl-1,3-dithiane derivatives) like energy level differences between the stable form to unstable form, bond length of C7–H8, C7–H1 (see Schemes 4 and 5 respectively) and C1–C7, dihedral angle of H1 and H8 with aromatic ring, Mullikan charges of H1 & H8, and vibrational frequencies of H1 and H8 were calculated as well. The results are demonstrated in the schemes and the related tables (Schemes 4 and 5, Tables 5 and 6).

## Results and discussion

To compare the efficiency of SA-MNPs catalyst for acetalization reaction, different organic and inorganic solid acid catalysts were examined upon the reaction of 4-nitrobenzaldehyde (0.151 g, 1 mmol) with ethylene glycol (0.11 mL, 2 mmol) (Table 1). Acidic solid salts Al(HSO_4_)_3_, Zn(HSO_4_)_2_, Fe(HSO_4_)_3_, Ca(HSO_4_)_2_ were tested in cyclic acetalization reaction and they had the yield about 80% but with (NH_4_)_2_SO_4_, the yield of the reaction strongly decreased (entries 1–5 in Table 1). Trityl (triphenylmethyl) chloride and trityl bromide were also examined as solid acids for described reaction and the desired product was obtained in 76% yield (entries 6 and 7). The reaction was performed in the presence of silica sulfuric acid and the yield of the reaction improved (entry 8), while the reaction was carried out with sulfamic acid the crude yield was 74% (entry 9). Hydroxylamine-*O*-sulfonic acid with acidic characteristic which is another inorganic compound, was also tested for acetalization of nitrobenzaldehyde and the yield of desired product decreased up to 57% (entry 10). Pyridinium hydrogen sulphate was synthesized using the reported procedure by Moosavi-Zare and co-workers.^27^ It was tested for this reaction and the desired product was obtained in 81% yield (entry 11). Employing 1,10-phenanthroline-based molten salt as a bifunctional sulfonic acid, which was synthesized by Babaee and co-workers has shown a yield about 62% (entry 12).^28^ The reaction failed in the absence of a catalyst (entry 13). The SA-MNPs catalyst was found to be the most effective solid acid for cyclic acetalization and thioacetalization. In order to study the best amount of SA-MNPs catalyst, it was examined 0.025, 0.05, 0.1 and 0.2 g of the catalyst (entries 14–17). Whenever the amount of catalyst was increased, the yield of desired product increased as well, but the excess amount of the catalyst caused a decrease in the reaction yield (entry 17). So, the amount of the catalyst should be optimized. The results demonstrated that 0.1 g of SA-MNPs catalyst with the yield of 95% was the optimum amount of catalyst for acetalization reaction (Table 1, entry 16).

**Table 1:** The reaction of 4-nitrobenzaldehyde (1 mmol) with ethylene glycol (2 mmol) in the presence of various catalysts at room temperature (25 °C) under solvent-free conditions for 1 hour

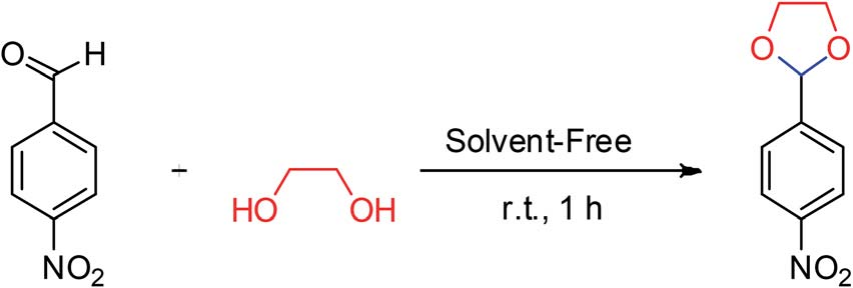			
Entry	Catalyst	Amount of cat. (g)	Yield (%)
1	Al(HSO_4_)_3_	0.2	81
2	Zn(HSO_4_)_2_	0.2	83
3	Fe(HSO_4_)_3_	0.2	82
4	Ca(HSO_4_)_2_	0.2	78
5	(NH_4_)_2_SO_4_	0.2	10
6	Trityl chloride	0.1^a^	76
7	Trityl bromide	0.12^a^	76
8	Silica sulfuric acid	0.2	87
9	Sulfamic acid	0.1	74
10	Hydroxylamine-*O*-sulfonic acid	0.1	57
11	Pyridinium hydrogen sulphate^b^	0.12^a^	81
12	1,10-Phenanthroline-based molten salt^c^	0.16	62
13	—	0.0	0
14	SA-MNPs	0.025	52
15	SA-MNPs	0.05	71
**16**	**SA-MNPs**	**0.1**	**95**
17	SA-MNPs	0.2	82

a 40 mol%.

b Ref. 27.

c Ref. 28.

Various organic and inorganic solid acid catalysts were tested for methoxymethylation in the reaction of benzyl alcohol (0.103 mL, 1 mmol) with the excess of formaldehyde dimethyl acetal (FDMA) (0.88 mL, 10 mmol) in order to have a comparison with the efficiency of SA-MNPs catalyst (Table 2). Hydrogen sulfate as solid acidic salts such as: Al(HSO_4_)_3_, Zn(HSO_4_)_2_, Fe(HSO_4_)_3_ and Ca(HSO_4_)_2_ were tested for methoxymethylation reaction and their yields are shown in Table 2 (entries 1–4). The organic solid Lewis acids, trityl chloride and trityl bromide were also utilized for methoxymethylation reaction and the desired product was obtained in 78% and 75% yields, respectively (entries 5 and 6). The reaction was done in the presence of silica sulfuric acid and had the yield of 90% (entry 7). When the reaction was accomplished with sulfamic acid, the yield was observed 81% (entry 8). Hydroxylamine-*O*-sulfonic acid was another organic solid acid, which was used for methoxymethylation and the desired product was obtained in 73% yield (entry 9). To find the optimum amount of catalyst for preparation of MOM-ethers, 0.025, 0.05, 0.1 g of SA-MNP catalyst were applied (entries 10–13). Whenever the amount of catalyst decreased, the yield of the desired product decreased as well, but the excess amount of catalyst had negative effect (entry 13). The results demonstrated that 0.1 g of SA-MNPs catalyst with the yield of 97% was the optimum amount for methoxymethylation reaction (Table 2, entry 12).

**Table 2:** The reaction of benzyl alcohol (1 mmol) with formaldehyde dimethyl acetal (FDMA) (10 mmol) in the presence of catalysts at room temperature (25 °C) under solvent-free conditions for 2 hours

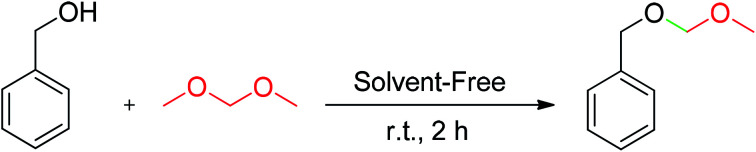			
Entry	Catalyst	Amounts of cat. (g)	Yield (%)
1	Al(HSO_4_)_3_	0.2	85
2	Zn(HSO_4_)_2_	0.2	81
3	Fe(HSO_4_)_3_	0.2	85
4	Ca(HSO_4_)_2_	0.2	83
5	Trityl chloride	0.1	78
6	Trityl bromide	0.1	75
7	Silica sulfuric acid	0.2	90
8	Sulfamic acid	0.1	81
9	Hydroxylamine-*O*-sulfonic acid	0.1	73
10	SA-MNPs	0.025	74
11	SA-MNPs	0.05	82
**12**	**SA-MNPs**	**0.1**	**97**
13	SA-MNPs	0.2	85

After the amount of SA-MNPs catalyst was optimized to protect carbonyl compounds at room temperature under solvent-free conditions (Table 1, entry 16), the efficiency of SA-MNPs was explored for the acetalization and thioacetalization reactions of different aromatic carbonyl derivatives with various diols and/or dithiol. The results are displayed in Table 3. The acetalization reaction with ethylene glycol *via* benzaldehyde derivatives were carried out (Table 3, 1a–1c). The acetalization process with pentaerythritol and 4-nitrobenzaldehyde and 3-nitrobenzaldehyde and 1-phenyl-1,2-ethanediol with 4-nitrobenzaldehyde were also performed under reflux conditions in acetonitrile for 6 hours with good yields (Table 3, 1e–1g). The thioacetalization reaction with 1,3-propanedithiol with benzaldehyde derivatives and 2-naphthaldehyde were achieved (Table 3, 2a–2h). Moreover, the products of 4-nitroacetophenone with ethylene glycol and 1,3-propanedithiol as examples of ketalization were occurred after 2 hours (Table 3, 1d, 2i). As Table 3 indicates, the aromatic aldehyde derivatives possessing electron-withdrawing and halogen substituents afforded the desired products with the short reaction times in excellent yields. Due to the characteristics of electron-withdrawing groups, the carbon of aldehydes is more positive and more susceptible of diol's attacking (Table 3, 1a–1c, 2a–2c), whereas the aldehyde with the electron-donating groups needed more time to complete the reaction (Table 3, 2d–2h).

**Table 3:** Conversion of carbonyl compounds to acetals and thioacetals using SA-MNPs at room temperature, under solvent-free conditions^a^

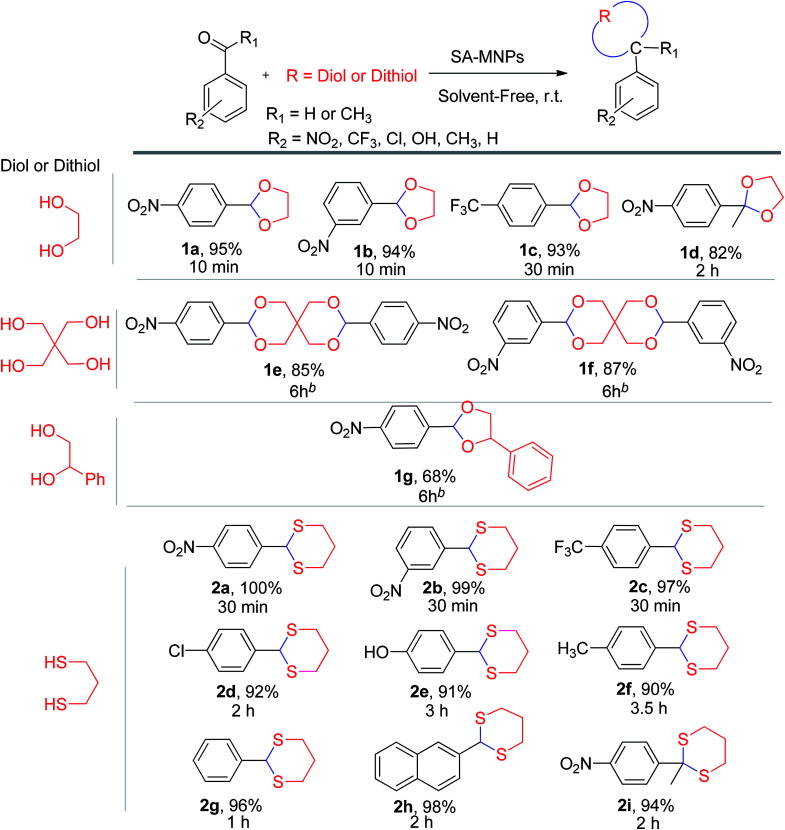

a Carbonyl compound (1 mmol), diol or dithiol (3 mmol) and SA-MNPs (0.1 g).

b Under reflux in acetonitrile.

After the optimization of the amount of SA-MNPs for methoxymethylation reaction at room temperature (25 °C) under solvent-free conditions (Table 2, entry 12), the efficiency of this catalyst was studied by the preparation of methoxymethyl ethers (MOM-ethers). The results are displayed in Table 4. Benzyl alcohol derivatives with electron-donating and halogen substituents, afforded the desired products with short reaction times in good to excellent yields (Table 4, 3a–3g). Also, alkyl alcohol such as *n*-butanol gave the corresponding product in good yield (Table 4, 3h). But, benzyl alcohol with electron withdrawing substituents and phenols as weak nucleophiles had no yield in methoxymethylation reaction (Table 4, 3i–3k).

**Table 4:** Methoxymethylation of alcohols with SA-MNPs under solvent-free conditions at room temperature^a^

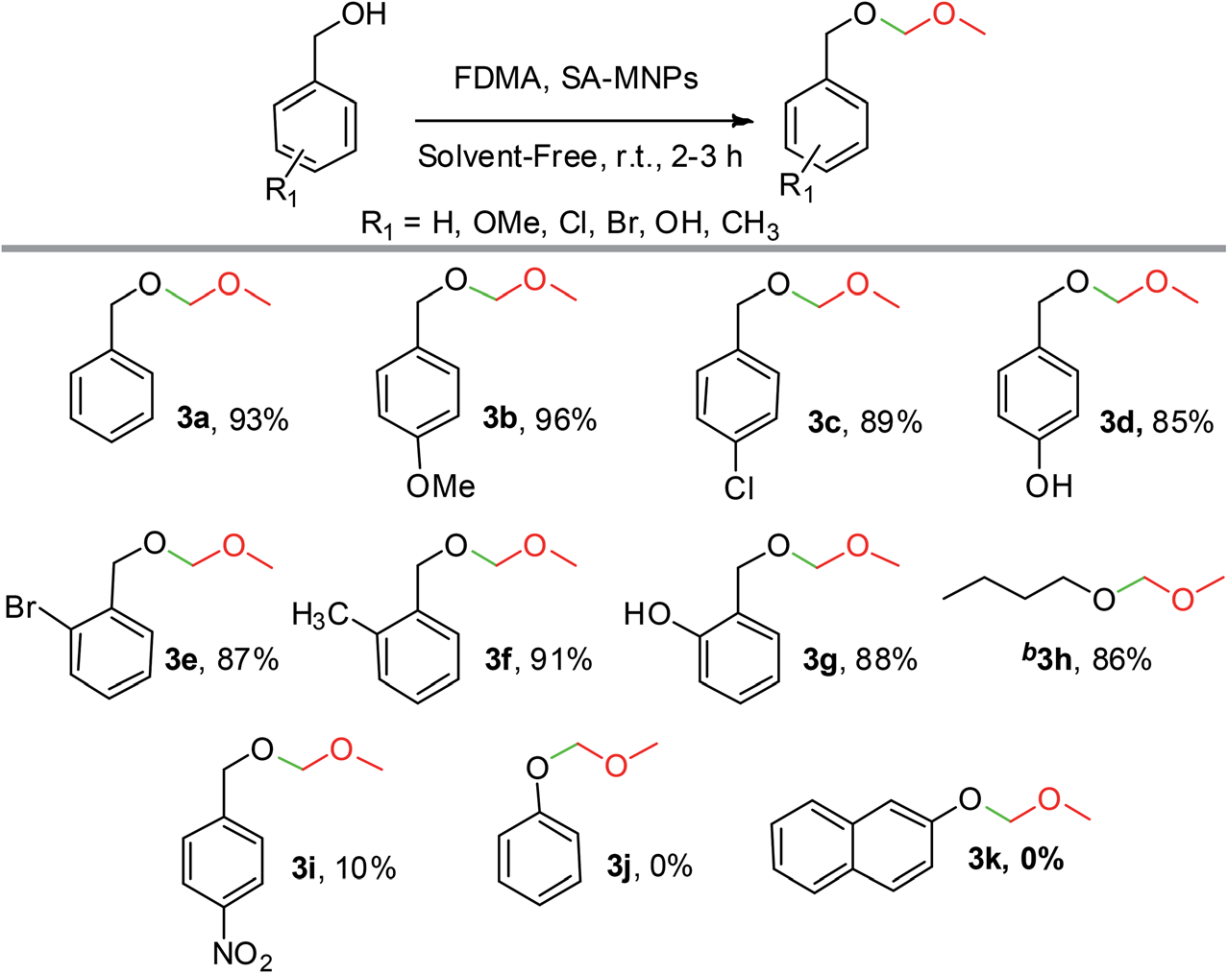

a Benzyl alcohol (1 mmol), formaldehyde dimethyl acetal (FDMA) (10 mmol, 0.88 mL) and SA-NMPs (0.1 g).

b *n*-Butanol is an example of alcohol has been participated in methoxymethylation reaction.

Cyclic acetals and thioacetals are suitable candidates for development of stereoelectronic effects.^29^ In continuation our previous computational investigations of stereoelectronic effects within the structures of suitable molecules,^30^ herein we wish to study the role of hyperconjugation and anomeric effect at the structures of desired products *via* computational calculations. The obtained theoretical results of the synthesized products (based on Tables 5 and 6) show different structural, stereochemistry and electronic properties of 2-phenyl-1,3-dioxolane derivatives in comparison with the 2-phenyl-1,3-dithiane derivatives, phenylcyclohexane and phenylcyclopentane molecules. In 2-phenyl-1,3-dioxolane derivatives H1 is nearly orthogonal toward aromatic ring (dihedral angle of H1 with aromatic ring [C3] in stable forms of derivatives is almost *θ* = 80°), whereas dihedral angle of H1 with aromatic ring [C3] in phenyl cyclopentane is almost *θ* = 0° (Scheme 4 and Table 5). It shows that maybe oxygen atoms have influence on stereo electronical properties of the structures.

**Table 5:** Selected structural data of stable forms of 2-phenyl-1,3-dioxolane derivatives

2-Phenyl-1,3-dioxolane derivatives (R=)	Energy difference between stable form to unstable form^a^ (kcal mol^−1^)	Dihedral angle of H1 with aromatic ring [C3] in stable form (*θ*°)	Bond length of C7–H1 (Å)	Bond length of C4–C7 (Å)	Mullikan charge of H1 (esu)	Frequency of H1 (cm^−1^)
(1a) NO_2_	3.80	81.50	1.099	1.526	0.161	3043
(1c) CF_3_	4.09	81.86	1.098	1.525	0.158	3049
Cl	4.01	80.04	1.098	1.524	0.156	3050
OH	3.99	78.86	1.098	1.522	0.150	3052
H	4.15	78.03	1.098	1.524	0.153	3051
CH_3_	4.03	77.58	1.098	1.523	0.151	3052
Phenyl cyclopentane^b^	1.21	0.93	1.099	1.515	0.125	3024

a In unstable form of 2-phenyl-1,3-dioxolane derivatives, dihedral angle of H8 with aromatic ring force to be almost *θ* = 0°.

b Red numbers related to phenylcyclopentane derivative model.

**Table 6:** Selected structural data of stable forms of 2-phenyl-1,3-dithiane derivatives

2-Phenyl-1,3-dithiane derivatives (R=)	Energy difference between stable form to unstable form^a^ (kcal mol^−1^)	Dihedral angle of H8 with aromatic ring [C3] in stable form (*θ*°)	Bond length of C7–H8 (Å)	Bond length of C4–C7 (Å)	Mullikan charge of H8 (esu)	Frequency of H8 (cm^−1^)
(2a) NO_2_	3.38	−0.37	1.094	1.510	0.193	3075
(2c) CF_3_	3.45	0.55	1.094	1.510	0.191	3075
(2d) Cl	3.42	0.00	1.094	1.510	0.190	3076
(2e) OH	3.65	0.00	1.094	1.509	0.187	3076
(2g) H	3.62	0.00	1.094	1.511	0.188	3079
(2f) CH_3_	3.64	0.14	1.094	1.510	0.188	3075
Phenyl cyclohexane^b^	3.02	−7.70	1.101	1.520	0.119	3006

a In unstable form of 2-phenyl-1,3-dithiane derivatives, dihedral angle of H8 with aromatic ring force to be almost *θ* = 90°.

b Red numbers related to phenyl cyclohexane derivative model.

Previous computational studies on 1,3-dioxolane's properties show that it is puckered molecule because of lone pairs, thus the geometries of 1,3-dioxolane's conformers do not seem to be appropriate for anomeric (n–σ*) interactions.^31^

**Scheme 6:**
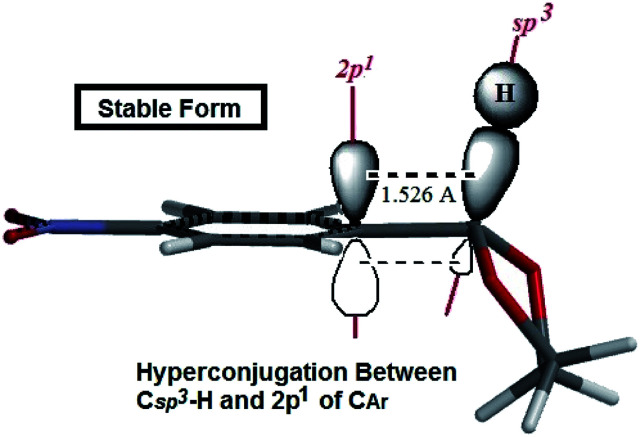
The predicted model of hyperconjugation between C–H and aromatic ring in 4NO_2_-2-phenyl-1,3-dioxolane.

**Scheme 7:**
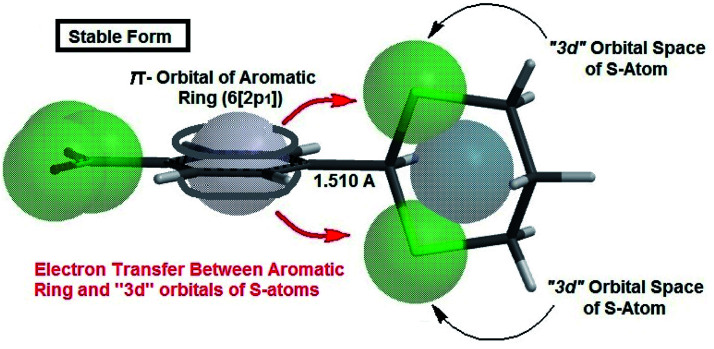
The predicted model of electron transfer between π-electrons of aromatic ring and 3d orbital space of S-atoms in 4NO_2_-2-phenyl-1,3-dithiane.

The authors believe that the hyperconjugation effect between C–H1 and aromatic ring (2p^1^ of C_Ar_) in diols has made this stable conformational structure and stereo electronical properties. Also, electron transfer between π-electrons of aromatic ring with 3d orbital space of S-atoms in the 2-phenyl-1,3-dithiane derivatives and electrostatic repulsion have made this stereochemistry. The predicted model of hyperconjugation between C–H and aromatic ring in one of the diol's derivatives and the predicted model of electron transfer between π-electrons of aromatic ring and 3d orbital space of S-atoms in one of the dithiol's derivatives have been modeled in Schemes 6 and 7 respectively.

### Spectral analysis to prove the predicted computational model

**Fig. 4:**
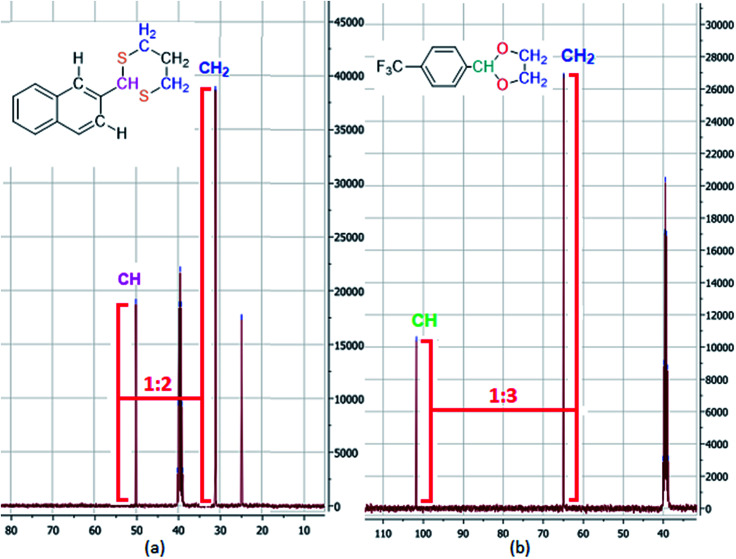
Comparing NOE effect in ^13^C-NMR spectrum of products. ((a) Part of ^13^C-NMR spectrum of 2-(naphthalen-2-yl)-1,3-dithiane (1c). (b) Part of ^13^C-NMR spectrum of 4CF_3_-2-phenyl-1,3-dioxolane (2h)).

In addition to computational study, in ^13^C-NMR spectrum of 4CF_3_-2-phenyl-1,3-dioxolane, the C–H to C–H_2_ ratio is approximately 1 : 3. But, in ^13^C-NMR spectrum of 2-(naphthalen-2-yl)-1,3-dithiane which is guessed to be influenced by NOE effect with CH aromatic ring, the C–H to C–H_2_ ratio is approximately 1 : 2. Therefore, the predicted model which claims that C–H of dithiane is parallel with C–H aromatic ring, could be abundant conformer of 2-(naphthalen-2-yl)-1,3-dithiane molecule (Fig. 4).

**Fig. 5:**
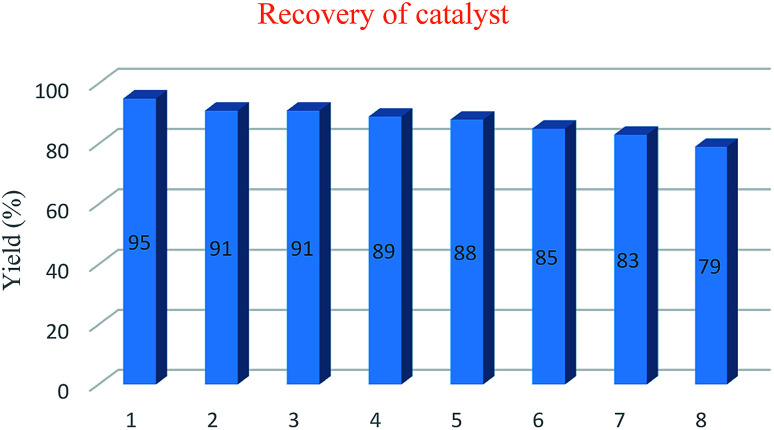
Reusability of the SA-MNPs catalyst for eight reaction runs.

The reusability of SA-MNPs catalyst was investigated for acetalization reaction of 4-nitrobenzaldehyde with ethylene glycol for eight runs under the optimized reaction conditions. The data are displayed in Fig. 5. The catalyst was separated from the reaction mixture by using an external magnet, repeatedly washed with di-isopropyl ether, dried and reused in the next run. It shows that the performance of the catalyst doesn't drop after eight sequential runs.

## Conclusion

Sulfamic acid-functionalized magnetic Fe_3_O_4_ nanoparticles (SA-MNPs) was applied in protection reactions of some carbonyl compounds with diols and dithiols and the reaction of alcohols with FDMA with good to excellent yields. Moreover, structural and electronic properties of some of the products were computed by DFT-B3LYP/6-31G* method. The experimental and theoretical investigation demonstrated that electronic effects like hyperconjugation and electron transfer, has made 2-phenyl-1,3-dioxolane and 2-phenyl-1,3-dithiane conformers become stabilized respectively. Also analysis of ^13^C-NMR spectrum of 2-(naphthalen-2-yl)-1,3-dithiane which is guessed to be influenced by NOE effect demonstrated that the predicted model which claims that C–H8 is almost parallel with C–H aromatic ring in 2-phenyl-1,3-dithiane derivatives, could be abundant conformers. The results show that the reusability of the SA-MNPs catalyst could be used for eight similar cycles of the reaction.

## Experimental

### General

All chemicals and solvents used in this article were purchased from Merck and Aldrich. The progress of the reaction and the purity of the products were determined using TLC performed with SIL G/UV 254 silica gel plates. All experiments were carried out under an atmosphere of air. ^13^C NMR spectra were reported in ppm relative to residual DMSO (39.52 ppm) that were obtained with ^1^H-decoupling and described in terms of chemical shift (*δ* in ppm). Data for ^1^H-NMR are described as following: chemical shift (*δ* in ppm), multiplicity (s, singlet; d, doublet; t, triplet; q, quartet; quin, quintet; sx, sextet; m, multiplet; app, apparent; br, broad signal), coupling constant (Hz), integration. NMR data were given in University of Isfahan (Bruker-400 MHz).

### General procedure for preparation of sulfamic acid-functionalized magnetic Fe_3_O_4_ nanoparticles (SA-MNPs)

**Scheme 8:**
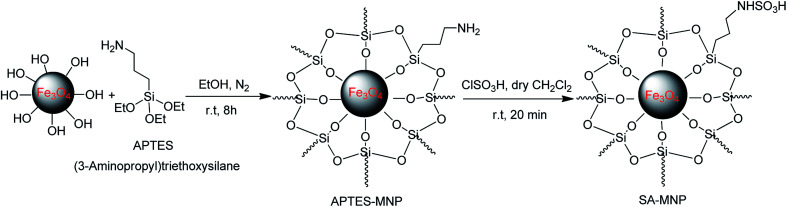
Synthesis of SA-MNPs according to the Kassaee and co-workers' reported method.^21*f*^

The SA-MNPs catalyst was synthesized by Kassaee and co-workers in 2011 and was produced according to their protocol and it is worthy to mention that, since the catalyst structure is similar to the previously reported catalyst, therefore, we did not characterize it again (Scheme 8).^21*f*^

### General procedure for synthesis of acetals and thioacetals from aldehyde derivatives

To a mixture of carbonyl compound (1 mmol) and SA-MNPs (0.1 g), diol or dithiol (3 mmol) was added and the mixture was stirred for a specific time under solvent-free conditions in a flask at room temperature. After consuming of carbonyl compound, as indicated by TLC, di-isopropyl ether was added and the nanomagnetic catalyst was separated with strong magnet and washed with dry CH_2_Cl_2,_ then the product was extracted with di-isopropyl ether and the organic solvent was evaporated to obtain desired product. In some cases, after evaporation of the solvent, the product has been purified by recrystallization with hot ethanol.

### General procedure for synthesis of methoxymethyl ether derivatives from alcohols

After adding alcohol (1 mmol) to a solution of formaldehyde dimethyl acetal (10 mmol) and SA-MNPs (0.1 g), the mixture was stirred at room temperature for 2–3 hours under solvent-free conditions in a round-bottom flask. Upon completion of alcohol as determined by TLC, diethyl ether (20 mL) was added to the vessel and the catalyst was removed by strong magnet and washed with CH_2_Cl_2_. Then, the product was purified by adding NaHCO_3_ solution (10% w/w) to the diethyl ether phase and decanted. The organic solvent was evaporated at room atmosphere.

## Conflicts of interest

There are no conflicts to declare.

## Supplementary Material

RA-010-D0RA09087E-s001
