# Supplementary material for: Catalytic application of sulfamic acid-functionalized magnetic Fe3O4 nanoparticles (SA-MNPs) for protection of aromatic carbonyl compounds and alcohols: experimental and theoretical studies
Source: RSC Adv. 2020 Dec 21;10(73):44946–57. doi: 10.1039/d0ra09087e (PMC9058591; doi:10.1039/d0ra09087e)
Supplement: RA-010-D0RA09087E-s001 [file RA-010-D0RA09087E-s001.pdf]

Electronic Supplementary Material (ESI) for RSC Advances.
This journal is © The Royal Society of Chemistry 2020

Supplementary data

Catalytic Application of Sulfamic Acid-Functionalized Magnetic Fe_3O_4 Nanoparticles (SA-MNPs) For Protection of Carbonyl Compounds and Alcohols; Experimental and Theoretical Studies

Sepideh Khaef,[a,b] Mohammad Ali Zolfigol,[a]* Avat Arman Taherpour,[b]* Meysam Yarie [a]

[a] Department of Organic Chemistry, Faculty of chemistry, Bu Ali Sina University, Hamedan, Iran

[b] Department of Organic Chemistry, Faculty of chemistry, Razi University, Kermanshah, Iran

*Email: zolfi@basu.ac.ir, mzolfigol@yahoo.com, and avat.taherpour@gmail.com

**Table of Contents**	
General Information	**S_2**
Experimental	**S_2**
Schemes of derivatives' stable forms	**S_3**
Physical properties and spectral analysis data	**S_5**
References	**S_8**
Spectra	**S_9**

General Information

All chemicals and solvents used in this article were purchased from Merck and Aldrich. The progress of the reaction and the purity of the products were determined using TLC performed with SIL G/UV 254 silica gel plates. All experiments were carried out under an atmosphere of air. ^{13}C NMR spectra were reported in ppm relative to residual DMSO (39.52 ppm) that were obtained with ^{1}H-decoupling and described in terms of chemical shift (δ in ppm). Data for ^{1}H-NMR are described as following: chemical shift (δ in ppm), multiplicity (s, singlet; d, doublet; t, triplet; q, quartet; quin, quintet; sx, sextet; m, multiplet; app, apparent; br, broad signal), coupling constant (Hz), integration. NMR data were given in University of Isfahan (Bruker-400 MHz).

Experimental

2-2-3 Preparation of sulfamic acid-functionalized magnetic Fe_3O_4 nanoparticles (SA-MNPs)

2-2-3-1 Preparation of MNPs coated by (3-aminopropyl)-triethoxysilane

After preparation of the magnetic Fe_3O_4 nanoparticles [1], the obtained MNPs powder (1.5 g) was dispersed in 150 mL ethanol by sonication for 15 min, then APTES (99%, 2.5 mL) was added to the mixture. After mechanical agitation under N_2 atmosphere at 40 °C for 4 h, the suspended substance was separated with centrifugation for 30 min. The settled product was re-dispersed in ethanol by sonication and then was isolated with magnetic decantation for 5 times. The precipitated product (APTES–MNPs) was dried at room temperature [2].

2-2-3-2 Preparation of sulfamic acid-functionalized magnetic Fe_3O_4 nanoparticles (SA-MNPs)

The APTES–MNPs (0.5 g) were dispersed in dry CH_2Cl_2 (3 mL) by ultrasonic bath for 10 min. Subsequently, chlorosulfuric acid (0.8 mL) was added dropwise over a period of 30 min at room temperature. Hydrogen chloride gas evolved from the reaction vessel immediately. Then, the prepared functionalized MNPs nanoparticles were separated by magnetic and washed three times with dry CH_2Cl_2 to remove the unattached substrates [2].

Schemes of derivatives' stable forms:

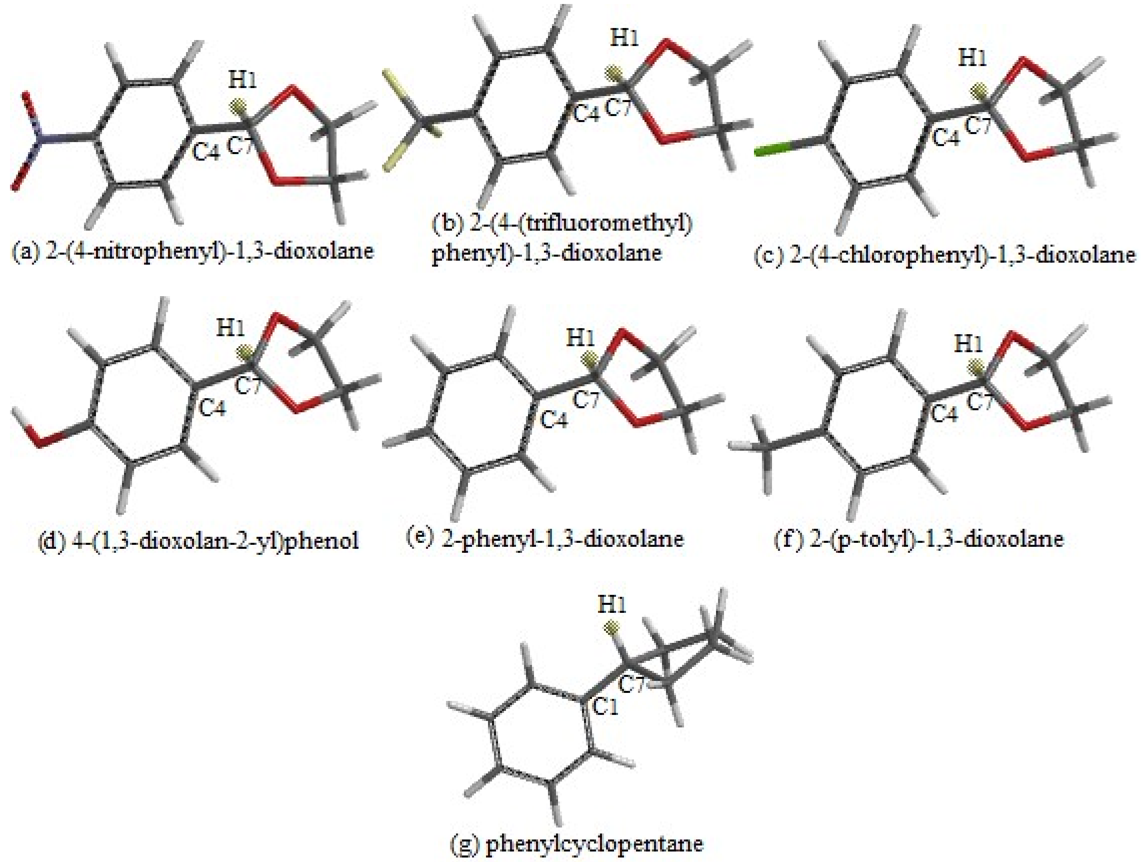

Scheme 1. Stable forms of 2-phenyl-1,3-dioxolane derivatives

Name	Structure	Optimized total energy:
(a) 2-(4-nitrophenyl)-1,3-dioxolane	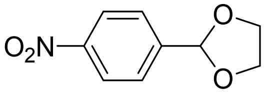	-703.903523 au
(b) 2-(4-(trifluoromethyl)phenyl)-1,3-dioxolane	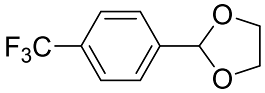	-836.439619 au
(c) 2-(4-chlorophenyl)-1,3-dioxolane	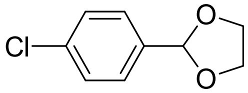	-958.997867 au
(d) 4-(1,3-dioxolan-2-yl)phenol	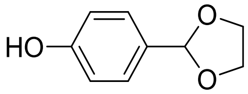	-574.617726 au
(e) 2-phenyl-1,3-dioxolane	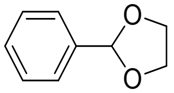	-499.401386 au
(f) 2-(p-tolyl)-1,3-dioxolane	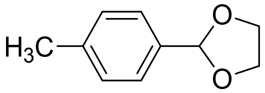	-538.719448 au

(g) phenyl cyclopentane	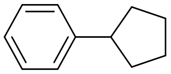	-427.607711 au

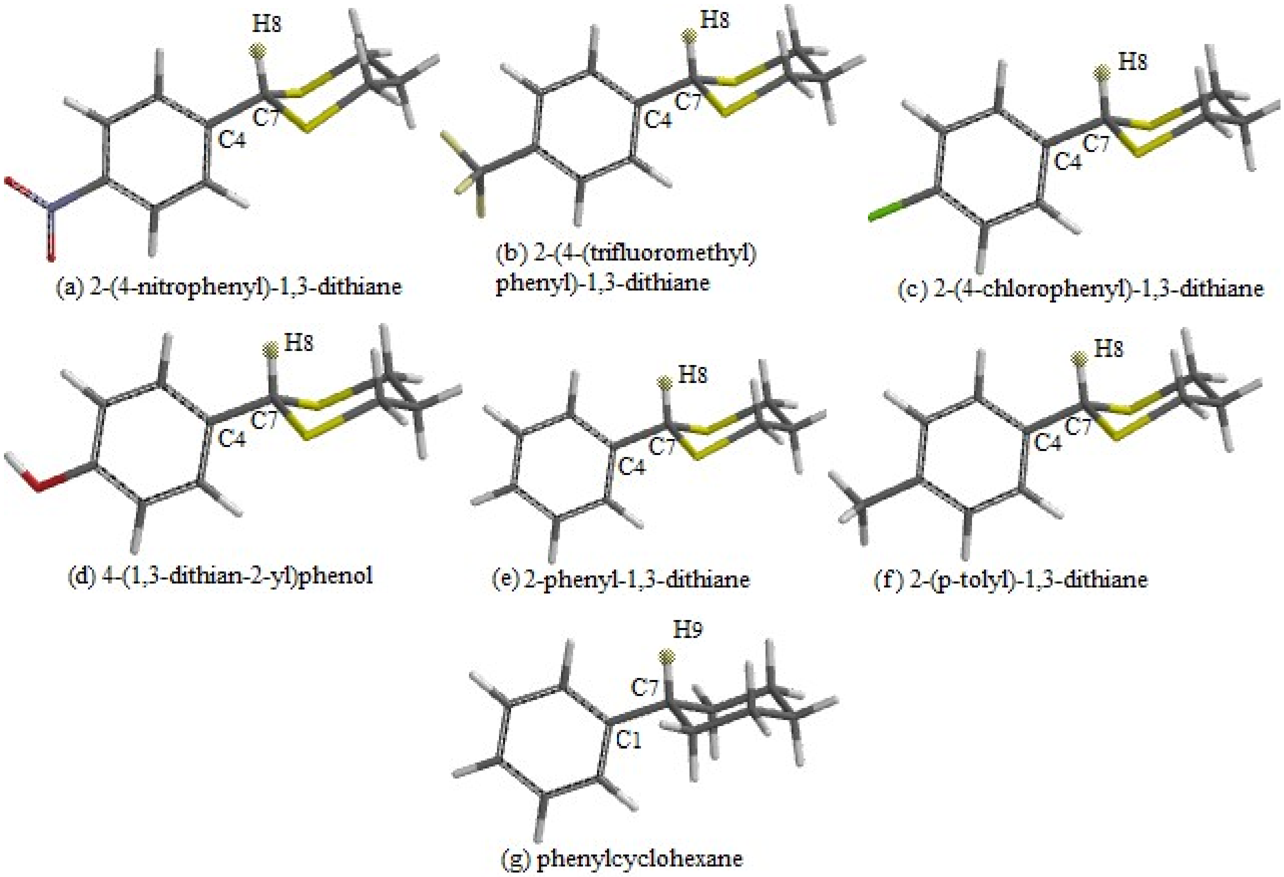

Scheme 2. Stable forms of 2-phenyl-1,3-dithiane derivatives

Name	Structure	Optimized total energy:
(a) 2-(4-nitrophenyl)-1,3-dithiane	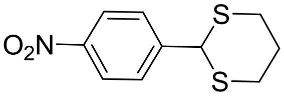	-1389.17299 au
(b) 2-(4-(trifluoromethyl)phenyl)-1,3-dithiane	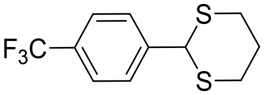	-1521.7093 au
(c) 2-(4-chlorophenyl)-1,3-dithiane	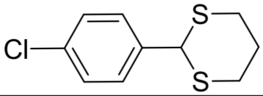	-1644.26761 au
(d) 4-(1,3-dithian-2-yl)phenol	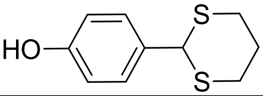	-1259.88822 au
(e) 2-phenyl-1,3-dithiane	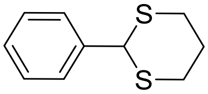	-1184.67172 au
(f) 2-(p-tolyl)-1,3-dithiane	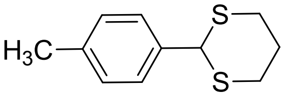	-1223.99008 au

(g) phenyl cyclohexane	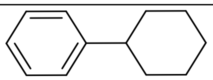	-466.930364 au

Physical properties and spectral analysis data:

*2-(4-nitrophenyl)-1,3-dioxolane (**1a**) (Table 3, row 1)* [3]

Creamy solid; Mp: 85-87 ˚C; IR (KBr, cm^{-1}): 3085, 2895, 1611, 1523, 1355, 1079, 847, 750.

*2-(3-nitrophenyl)-1,3-dioxolane (**1b**) (Table 3, row 1)* [4]

Yellow solid; Mp: 54 ˚C; IR (KBr, cm^{-1}): 2920, 1530, 1348, 1260, 1097, 1022, 803.

*2-(4-(trifluoromethyl)phenyl)-1,3-dioxolane (**1c**) (Table 3, row 1)* [5]

White solid; Mp: 44 ˚C; IR (KBr, cm^{-1}): 2894, 1433, 1329, 1164, 1117, 836. ^{1}H NMR (400 MHz, DMSO-d_6) δ_{ppm}: 7.69 (d, J = 8.0 Hz, 2H), 7.58 (d, J = 8.0 Hz, 2H), 5.77 (s, 1H), 4.02 – 3.85 (m, 4H). ^{13}C NMR (101 MHz, DMSO) δ_{ppm}: 142.65, 130.07, 129.63, 129.32, 127.33, 125.2 (q), 101.73, 64.93.

*2-methyl-2-(4-nitrophenyl)-1,3-dioxolane (**1d**) (Table 3, row 1)* [6]

White solid; Mp: 72-73 ˚C; IR (KBr, cm^{-1}): 3109, 2974, 2900, 1608, 1521, 1346, 1032, 883, 703.

*3,9-bis(4-nitrophenyl)-2,4,8,10-tetraoxaspiro[5.5]undecane (**1e**) (Table 3, row 2)* [7]

Yellow solid; Mp: 224-225 ˚C; IR (KBr, cm^{-1}): 2918, 2849, 1525, 1347, 1260, 1095, 1018, 799.

*3,9-bis(3-nitrophenyl)-2,4,8,10-tetraoxaspiro[5.5]undecane (**1f**) (Table 3, row 2)* [7]

White solid; Mp: 116 ˚C; IR (KBr, cm^{-1}): 2950, 2868, 1536, 1348, 1163, 1069, 902, 740. ^{1}H NMR (400 MHz, DMSO-d6) δ_{ppm}: 8.47 – 8.16 (m, 4H), 7.93 (dt, J = 7.8, 1.3 Hz, 2H), 7.72 (dd, J = 9.0, 7.7 Hz, 2H), 5.71 (s, 2H), 4.61 (dd, J = 11.3, 2.3 Hz, 2H), 4.00 (d, 2H), 3.90 (dd, J = 11.5, 2.4 Hz, 2H), 3.76 (d, J = 11.6 Hz, 2H). ^{13}C NMR (101 MHz, DMSO) δ_{ppm}: 147.53, 139.98, 132.89, 129.99, 123.80, 120.76, 99.43, 69.86, 69.34, 32.12.

*2-(4-nitrophenyl)-4-phenyl-1,3-dioxolane (**1g**) (Table 3, row 3)* [8]

White solid; Mp: 76 ˚C; IR (KBr, cm^{-1}): 2876, 1608, 1523, 1349, 1216, 1094, 854, 699. ^{1}H NMR (400 MHz, DMSO-d6) δ_{ppm}: 8.31 (d, 2H), 7.81 (d, 2H), 7.66 – 7.23 (m, 5H), 6.33 (s, 1H), 5.26 (dd, J = 7.5, 6.2 Hz, 1H), 4.57 (dd, J = 8.4, 6.3 Hz, 1H), 3.82 (t, J = 8.4, 7.6 Hz, 1H). ^{13}C NMR (101 MHz, DMSO) δ_{ppm}: 147.93, 145.34, 139.15, 128.51, 128.05, 127.89, 126.25, 123.59, 102.35, 77.51, 72.26.

*2-(4-nitrophenyl)-1,3-dithiane (**2a**) (Table 3, row 4)* [9]

White solid; Mp: 131-132 ˚C; IR (KBr, cm^{-1}): 3075, 2908, 1604, 1519, 1342, 857, 729.

*2-(3-nitrophenyl)-1,3-dithiane (**2b**) (Table 3, row 4)* [10]

White solid; Mp: 110-111 ˚C; IR (KBr, cm^{-1}): 2913, 1528, 1350, 1090, 725.

*2-(4-(trifluoromethyl)phenyl)-1,3-dithiane (**2c**) (Table 3, row 4)* [11]

White solid; Mp: 118 ˚C; IR (KBr, cm^{-1}): 2900, 1614, 1328, 1107, 1067, 863, 769.

*2-(4-chlorophenyl)-1,3-dithiane (**2d**) (Table 3, row 5)* [11]

Colorless solid; Mp: 88 ˚C; IR (KBr, cm^{-1}): 2929, 1488, 1404, 1275, 1084, 1013, 760, 673, 508.

*4-(1,3-dithian-2-yl)phenol (**2e**) (Table 3, row 5)* [9]

White solid; Mp: 153˚C; IR (KBr, cm^{-1}): 3369, 2887, 1597, 1511, 1427, 1242, 850, 763, 526.

*2-(p-tolyl)-1,3-dithiane (**2f**) (Table 3, row 5)* [12]

White solid; Mp: 81 ˚C; IR (KBr, cm^{-1}): 2932, 2892, 1602, 1511, 1421, 1275, 1168, 755, 674, 504.

*2-phenyl-1,3-dithiane (**2g**) (Table 3, row 6)* [10]

Pink solid; Mp: 66 ˚C; IR (KBr, cm^{-1}): 2890, 1451, 14161275, 1177, 730, 696.

*2-(naphthalen-2-yl)-1,3-dithiane (**2h**) (Table 3, row 6)* [9]

White solid; Mp: 111-112 ˚C; IR (KBr, cm^{-1}): 2893, 1628, 1598, 1505, 1420, 1278, 760, 476. ^{1}H NMR (400 MHz, DMSO-d_6) δ_{ppm}: 7.94 – 7.74 (m, 4H), 7.57 – 7.35 (m, 3H), 5.49 (s, 1H), 3.06 (ddd, J = 14.7, 12.5, 2.3 Hz, 2H), 2.85 (ddd, J = 14.2, 4.2, 2.8 Hz, 2H), 2.08 (ddt, J = 14.1, 4.5, 2.2

Hz, 1H), 1.76 – 1.58 (m, 1H). ^{13}C NMR (101 MHz, DMSO) δ 137.03, 132.74, 132.59, 128.29, 127.81, 127.53, 126.47, 126.41, 126.27, 125.62, 50.06, 31.03, 24.78.

*2-methyl-2-(4-nitrophenyl)-1,3-dithiane (**2i**) (Table 3, row 6)* [13]

White solid; Mp: 119-120 ˚C; IR (KBr, cm^{-1}): 2942, 1590, 1511, 1350, 1074, 852, 697.

*((methoxymethoxy)methyl)benzene (**3a**) (Table 4, row 1)* [14]

Yellow liquid; IR (KBr, cm^{-1}): 3031, 2882, 1496, 1454, 1379, 1104, 735, 697.

*1-methoxy-4-((methoxymethoxy)methyl)benzene (**3b**) (Table 4, row 1)* [15]

Oil; IR (KBr, cm^{-1}): 2930, 2836, 1610, 1500, 1247, 1107, 1033, 808.

*1-chloro-4-((methoxymethoxy)methyl)benzene (**3c**) (Table 4, row 1)* [16]

Yellow oil; IR (KBr, cm^{-1}): 2940, 2885, 1598, 1492, 1408, 1169, 1047, 804.

*4-((methoxymethoxy)methyl)phenol (**3d**) (Table 4, row 1)* [17]

Colorless solid; IR (KBr, cm^{-1}): 3501, 2964, 2926, 1604, 1474, 1223, 1081, 1013, 794, 472.

*1-bromo-2-((methoxymethoxy)methyl)benzene (**3e**) (Table 4, row 2)* [18]

Yellow liquid; IR (KBr, cm^{-1}): 2929, 2885, 1569, 1440, 1103, 1026, 748.

1-((methoxymethoxy)methyl)-2-methylbenzene (**3f**) *(Table 4, row 2)* [19]

Yellow liquid; IR (KBr, cm^{-1}): 2925, 1461, 1376, 1260, 1098, 1040, 743.

*2-((methoxymethoxy)methyl)phenol (**3g**) (Table 4, row 2)* [20]

Pink solid; Mp: 134 ˚C; IR (KBr, cm^{-1}): 3429, 2926, 1612, 1502, 1235, 1178, 1069, 1008, 886, 577, 455.

*1-(methoxymethoxy)butane (**3h**) (Table 4, row 2)* [14]

Yellow liquid; IR (KBr, cm^{-1}): 2960, 1228, 1174, 1068, 1017, 881, 851, 581.

References

1. M. A. Zolfigol, R. Ayazi-Nasrabadi, S. Baghery, Appl. *Organomet. Chem.,* 2016, **30**, 273–281.
2. M. Z. Kassaee, H. Masrouri, F. Movahedi, *Appl. Catal. A: General*, 2011, **395**, 28–33.
3. D. J. M. Lyons, R. D. Crocker, D. Endersb, T. V. Nguyen, *Green Chem.,* 2017, **19**, 3993-3996.
4. G. Xu, J. Cao, Y. Zhao, L. Zheng, M. Tao, W. Zhang, *Chem. Asian J.*, 2017, **12**, 2565 – 2575.
5. H. Yi, L. Niu, S. Wang, T. Liu, A. K. Singh, A. Lei, *Org. Lett.,* 2017, **19**, 122−125.
6. U. Azzena, M. Carraro, A. D. Mamuye, I. Murgiaa, L. Pisanoa, G. Zedde, *Green Chem.,* 2015, **17**, 3281-3284.
7. Y. Wang, X. Gong, Z. Wang, L. Dai, *J. Mol. Catal. A: Chem.,* 2010, **322**, 7–16.
8. S. Gouse, N. R. Reddy, S. Baskaran, *Org. Lett.,* 2019, **21**, 3822-3827.
9. K. Inamoto, T. Yamada, S. Kato, S. Kikkawa, Y. Kondo, *Tetrahedron,* 2013, **69**, 9192-9199.
10. B. Karami, M. Taei, S.Khodabakhshi, M. Jamshidi, *J. Sulfur Chem.,* 2012, **33**, 65–74.
11. Z. Xing, M. Yang, H. Sun, Z. Wang, P. Chen, L. Liu, X. Wang, X. Xie, X. She, *Green Chem.,* 2018, **20**, 5117-5122.
12. K. Nishino, K. Minato, T. Miyazaki, Y. Ogiwara, N. Sakai, *J. Org. Chem.,* 2017, **82**, 3659−3665.
13. T. Liu, L. Tian, J. Lai, D. Min, M. Qu, S. Tang, *Org. Biomol. Chem.,* 2017, **15**, 4068-4071.
14. T-S. Jin, J-J. Guo, Y-H. Yin, S-L. Zhang, T-S. Li, *J. Chem. Research (S),* 2002, **2002**, 188–189.
15. U. Azzena, L. Pisano, S. Mocci, *J. Organomet. Chem*., 2009, **694**, 3619–3625.
16. M. Moghadam, S. Tangestaninejad, V. Mirkhani, I. Mohammadpoor-Baltork, M. Khajehzadeh, *Monatsh Chem.,* 2010, **141**, 641–647.
17. B. V. Elgersma, R. Christiaan, h. Tijl, R. G. Elisabeth, Synthon biopharmaceuticals WO2018/69375, 2018, A1S.
18. P. Runyon, P. D. Mosier, B. L. Roth, R. A. Glennon, R. B. Westkaemper, *J. Med. Chem.*, 2008, **51**, 6808–6828.
19. M. F. Shostakovskii, B. A. Trofimov, A. S. Atavin, V. I. Lavrov, *Russ. Chem. Rev.,* 1968, 37.
20. M. Moghadam, S. Tangestaninejad, V. Mirkhani, I. Mohammadpoor-Baltork, M. Khajehzadeh, F. Kosari, M. Araghi, *Polyhedron,* 2010, **29**, 238–243.

Spectra: IR of all products, ^{1}H-NMR and ^{13}C-NMR spectra of some products

2-(4-nitrophenyl)-1,3-dioxolane (1a)

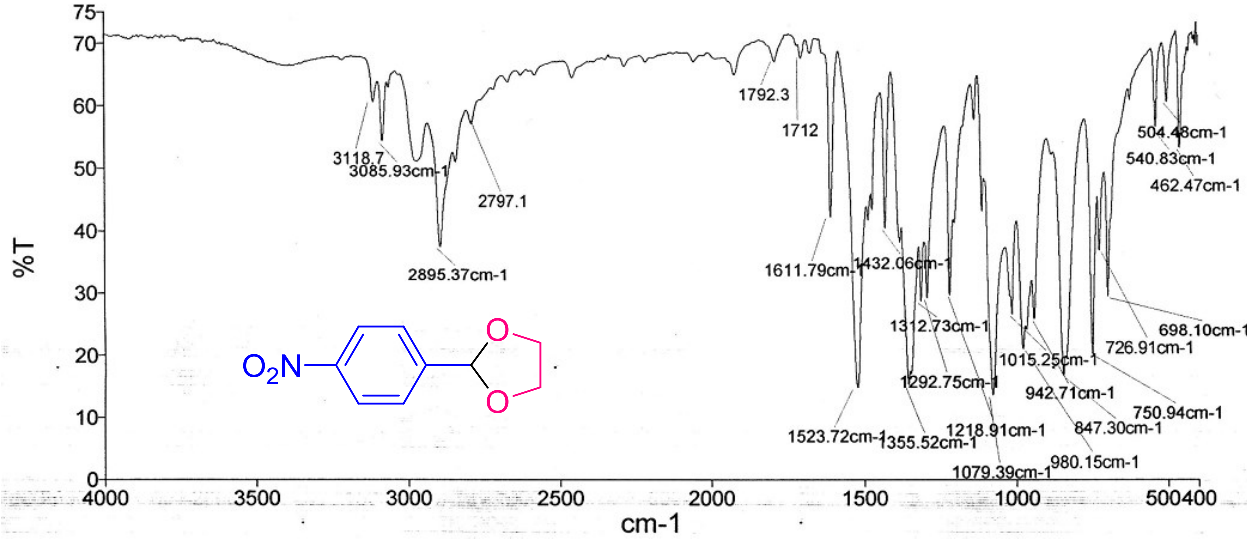

Figure 35. FT-IR spectrum of 2-(4-nitrophenyl)-1,3-dioxolane. (1a)

2-(3-nitrophenyl)-1,3-dioxolane (1b)

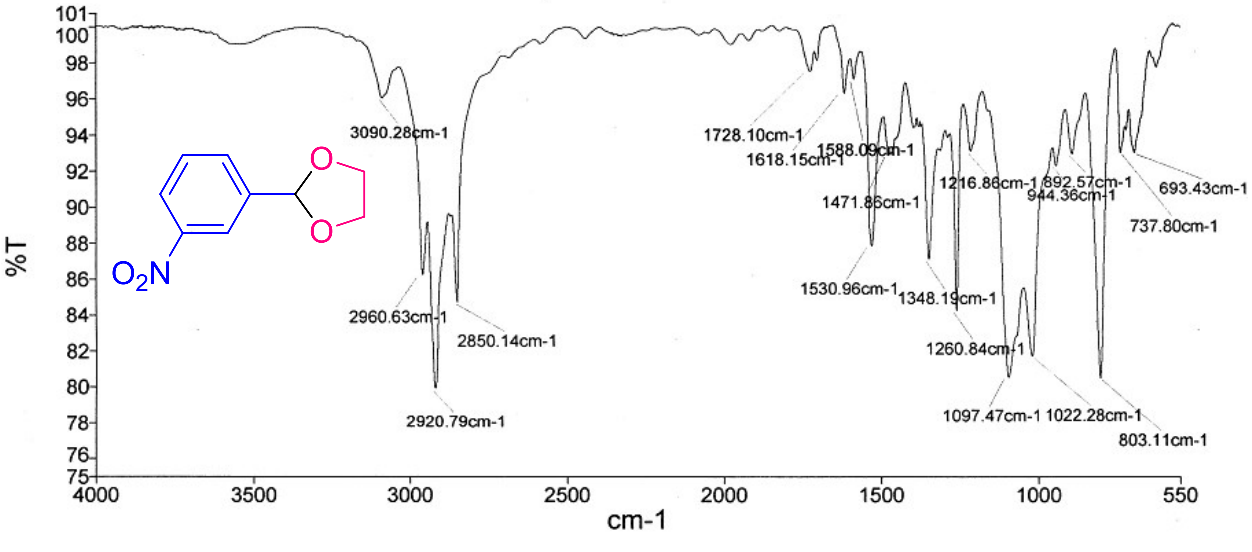

Figure 36. FT-IR spectrum of 2-(3-nitrophenyl)-1,3-dioxolane (1b)

2-(4-(trifluoromethyl)phenyl)-1,3-dioxolane (1c)

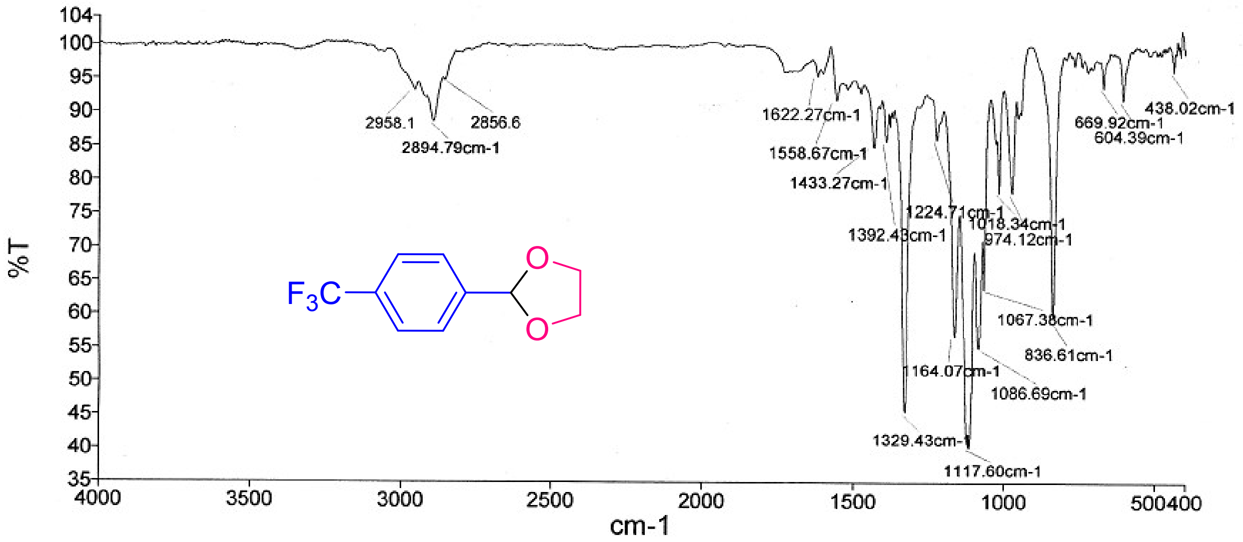

Figure 37. FT-IR spectrum of 2-(4-(trifluoromethyl)phenyl)-1,3-dioxolane (1c)

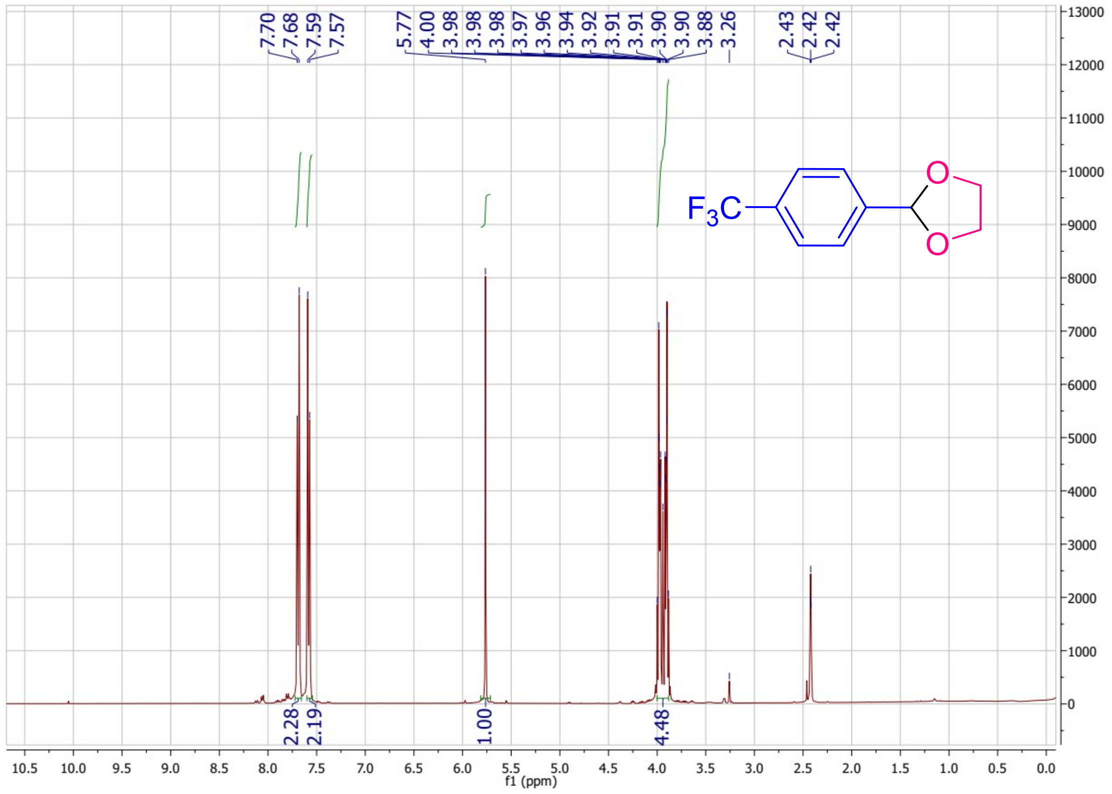

Figure 38. ^{1}H NMR spectrum of 2-(4-(trifluoromethyl)phenyl)-1,3-dioxolane (1c)

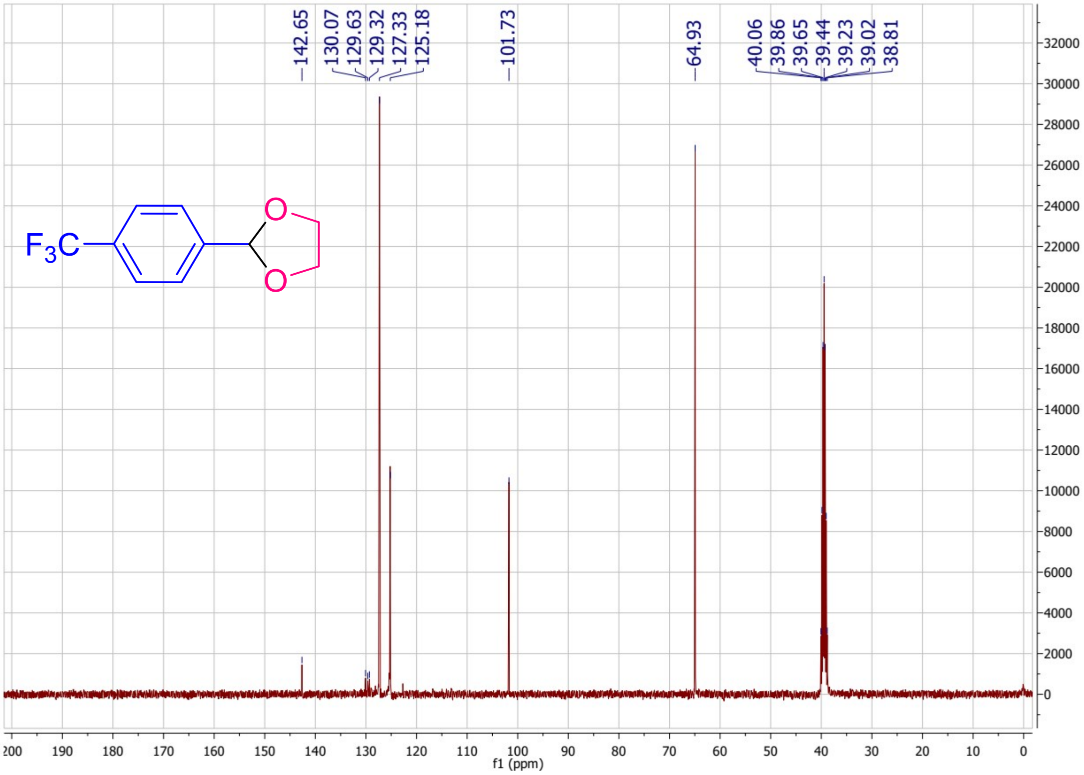

Figure 39. ^{13}C NMR spectrum of 2-(4-(trifluoromethyl)phenyl)-1,3-dioxolane (1c)

2-methyl-2-(4-nitrophenyl)-1,3-dioxolane (1d)

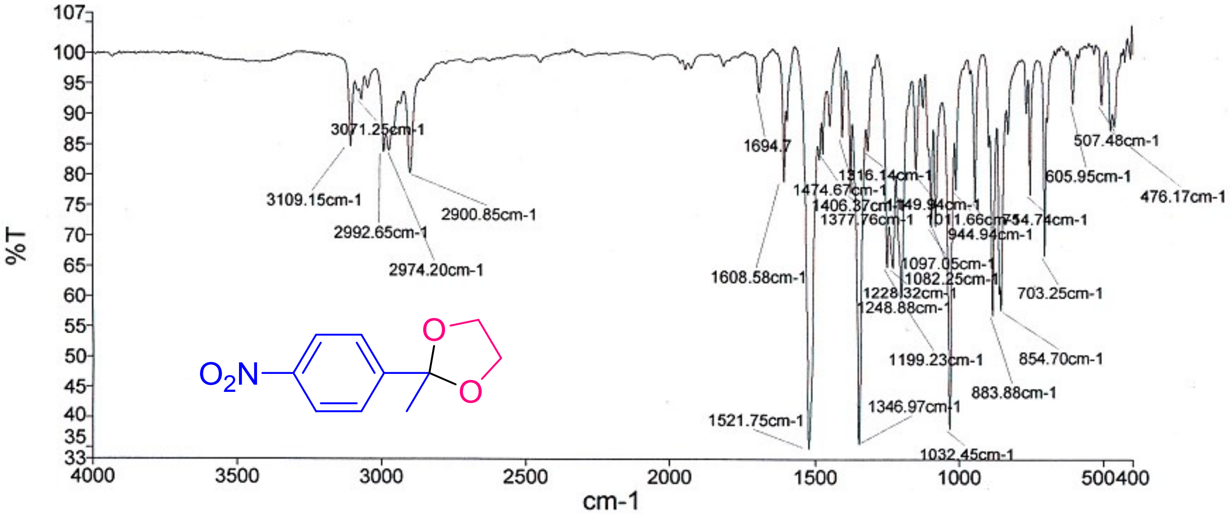

Figure 40. FT-IR spectrum of 2-methyl-2-(4-nitrophenyl)-1,3-dioxolane (1d)

3,9-bis(4-nitrophenyl)-2,4,8,10-tetraoxaspiro[5.5]undecane (1e)

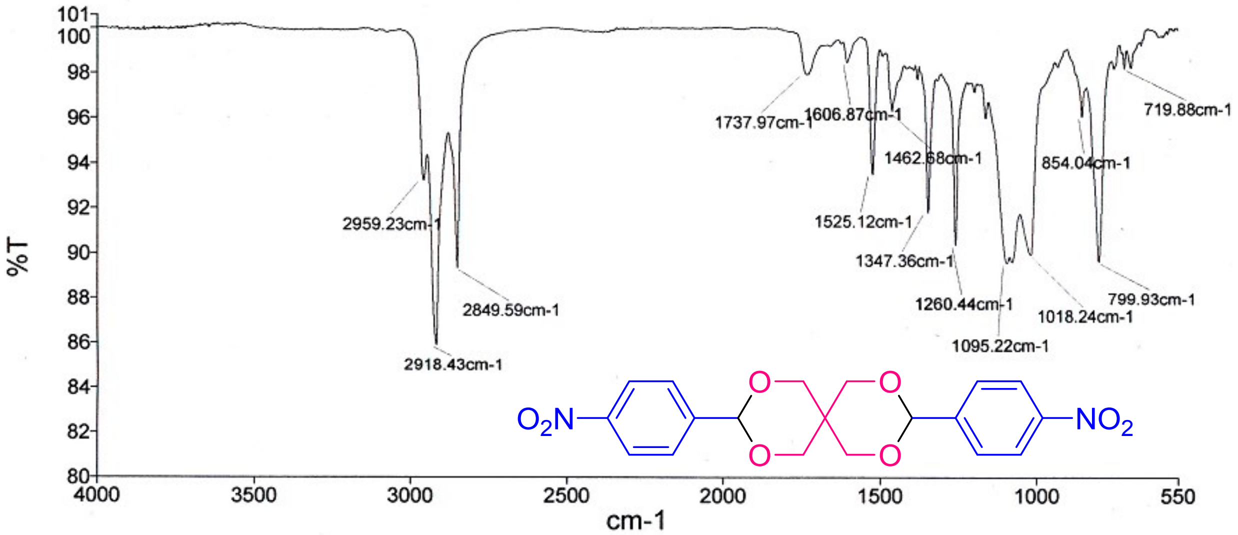

Figure 41. FT-IR spectrum of 3,9-bis(4-nitrophenyl)-2,4,8,10-tetraoxaspiro[5.5]undecane (1e)

3,9-bis(3-nitrophenyl)-2,4,8,10-tetraoxaspiro[5.5]undecane (1f)

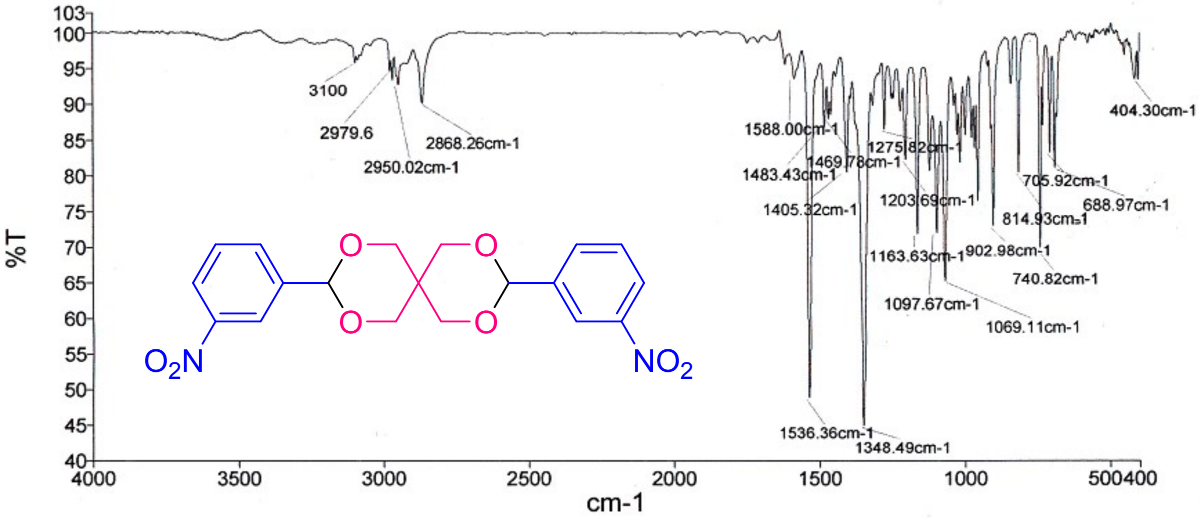

Figure 42. FT-IR spectrum of 3,9-bis(3-nitrophenyl)-2,4,8,10-tetraoxaspiro[5.5]undecane (1f)

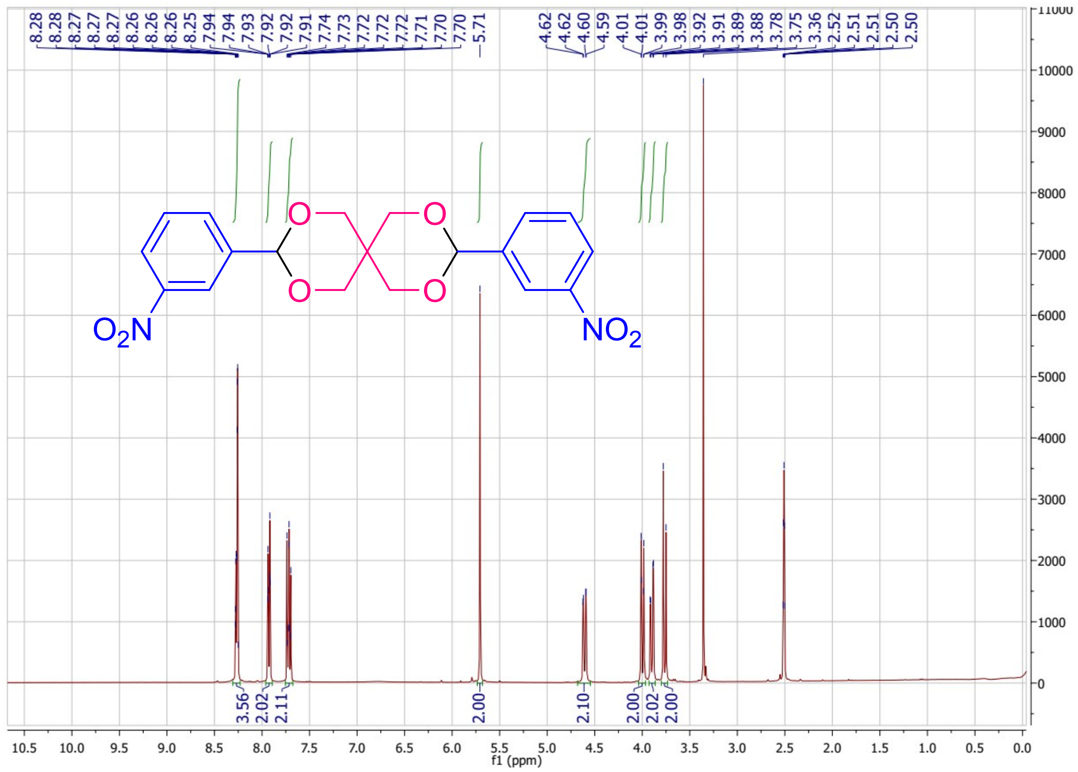

Figure 43. ^{1}H NMR spectrum of 3,9-bis(3-nitrophenyl)-2,4,8,10-tetraoxaspiro[5.5]undecane (1f)

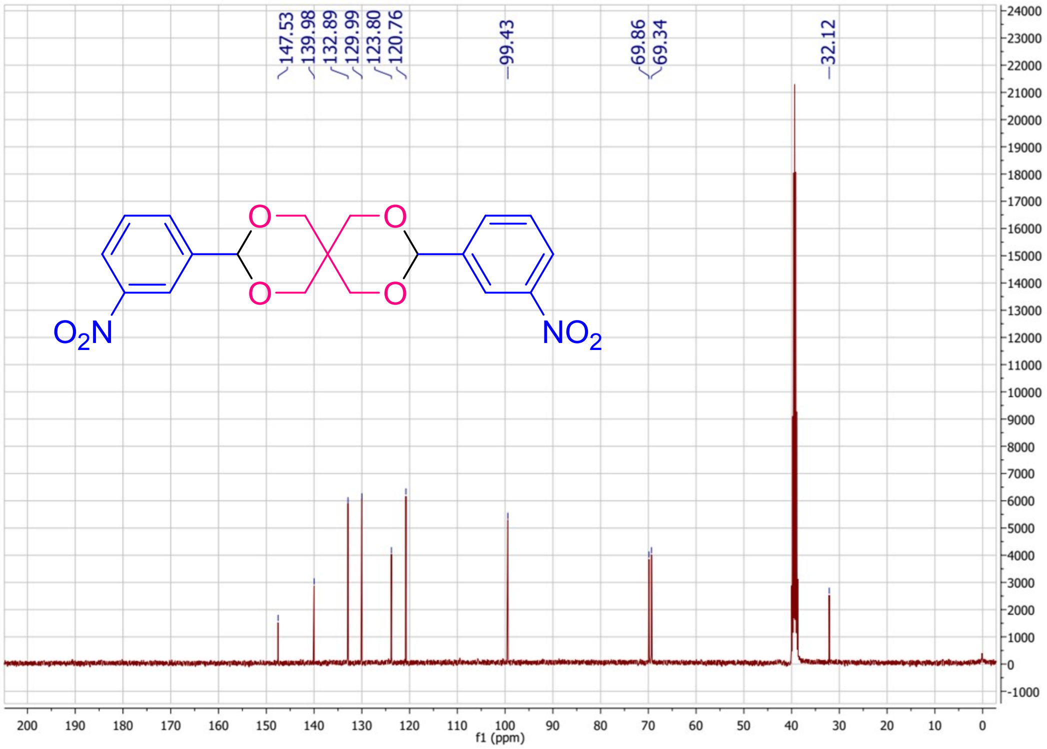

Figure 44. ^{13}C NMR spectrum of 3,9-bis(3-nitrophenyl)-2,4,8,10-tetraoxaspiro[5.5]undecane (1f)

2-(4-nitrophenyl)-4-phenyl-1,3-dioxolane (1g)

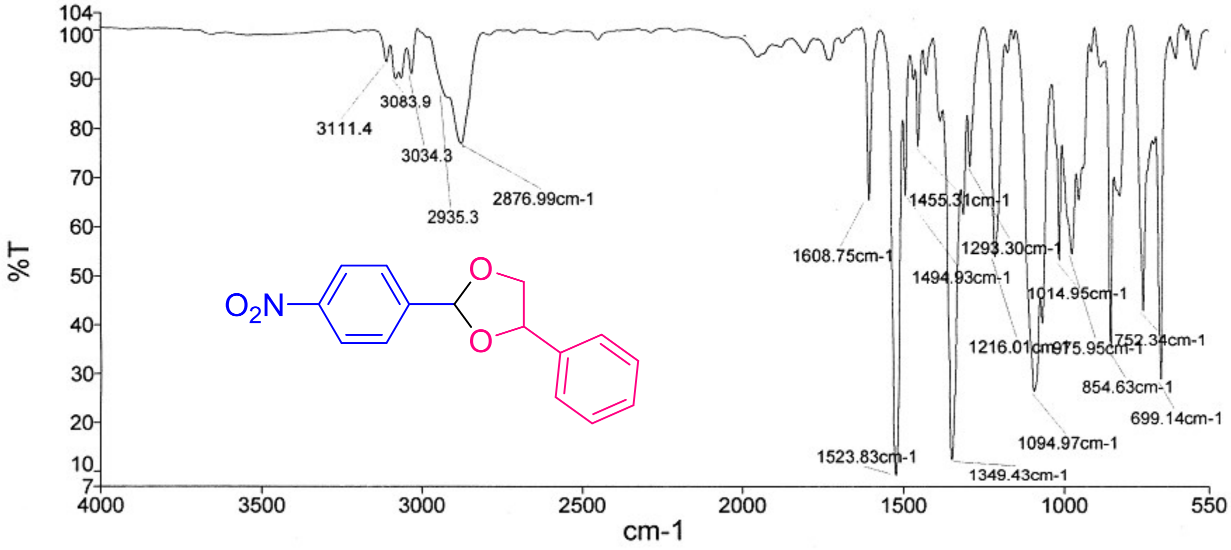

Figure 45. FT-IR spectrum of 2-(4-nitrophenyl)-4-phenyl-1,3-dioxolane (1g)

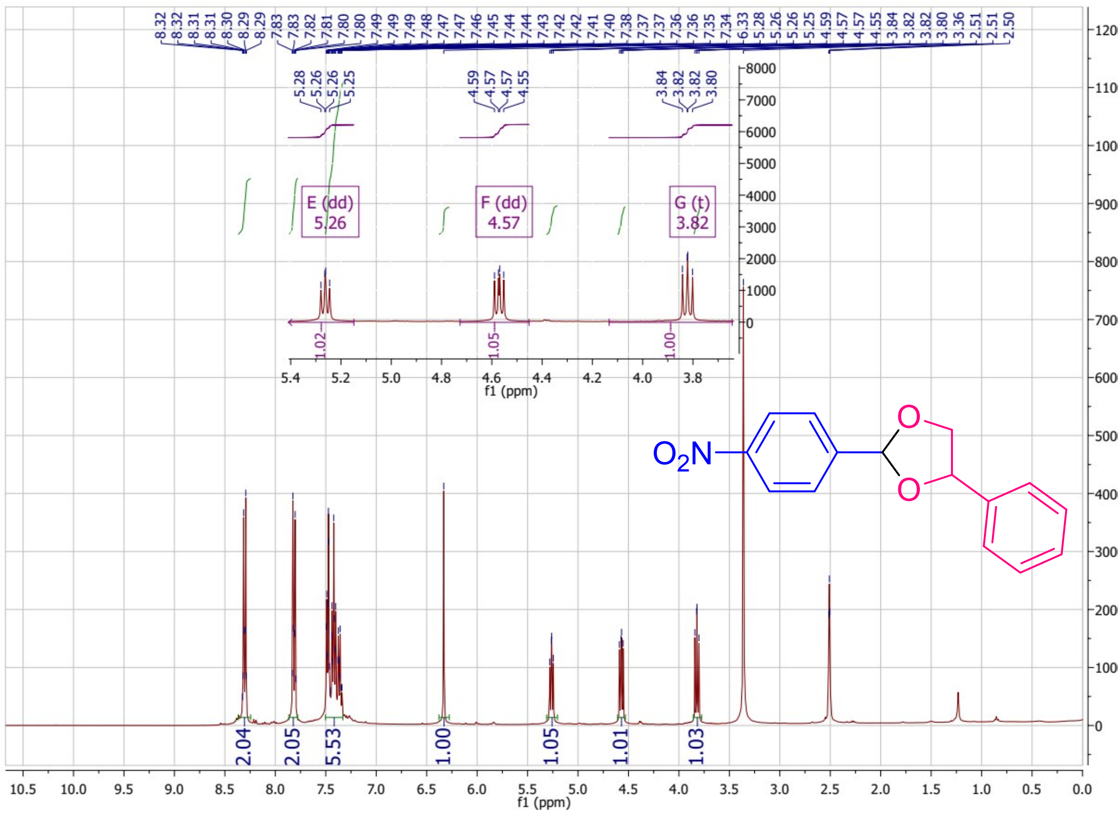

Figure 46. ^{1}H NMR spectrum of 2-(4-nitrophenyl)-4-phenyl-1,3-dioxolane (1g)

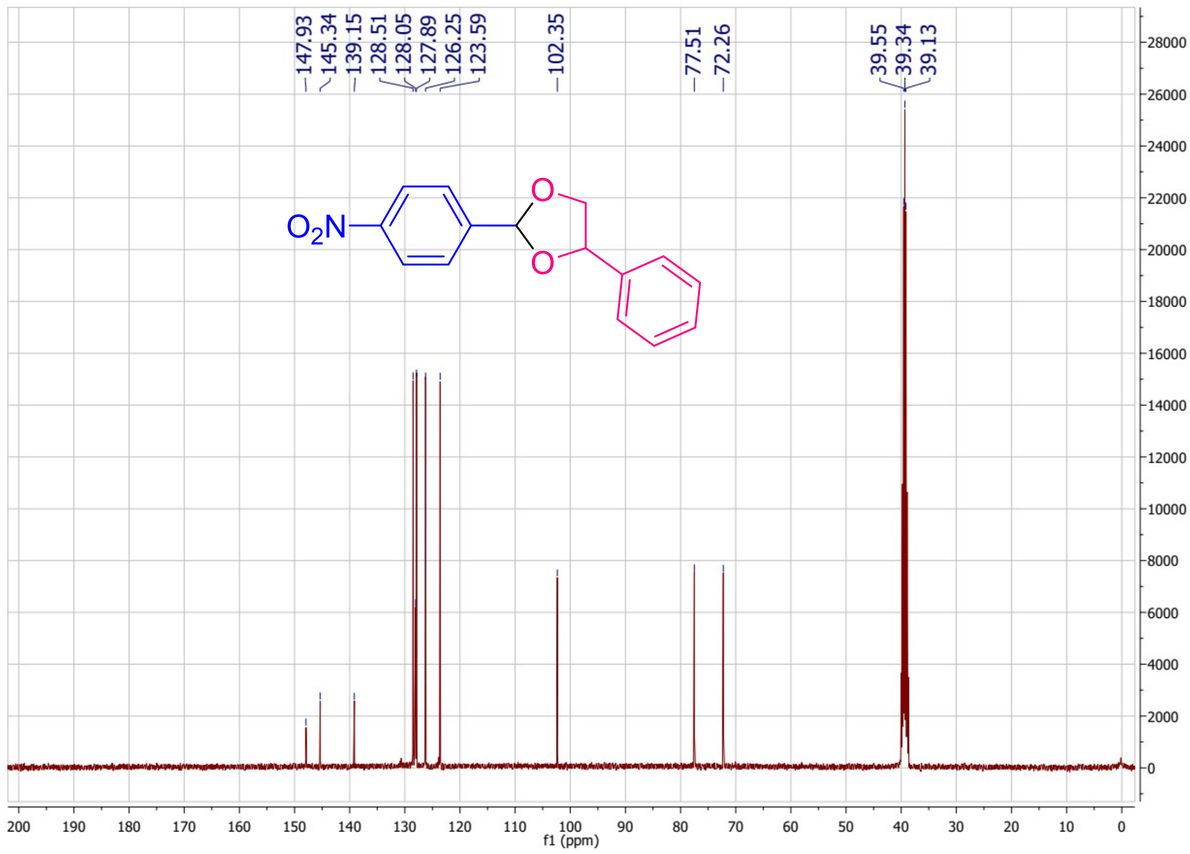

Figure 47. ^{13}C NMR spectrum of 2-(4-nitrophenyl)-4-phenyl-1,3-dioxolane (1g)

2-(4-nitrophenyl)-1,3-dithiane (2a)

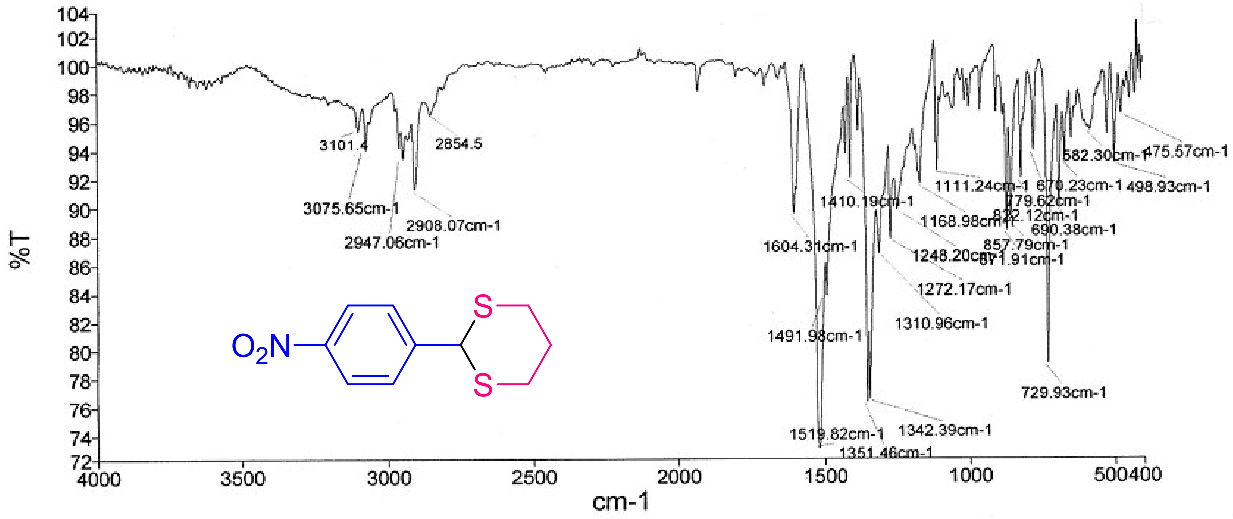

Figure 48. FT-IR spectrum of 2-(4-nitrophenyl)-1,3-dithiane (2a)

2-(3-nitrophenyl)-1,3-dithiane (2b)

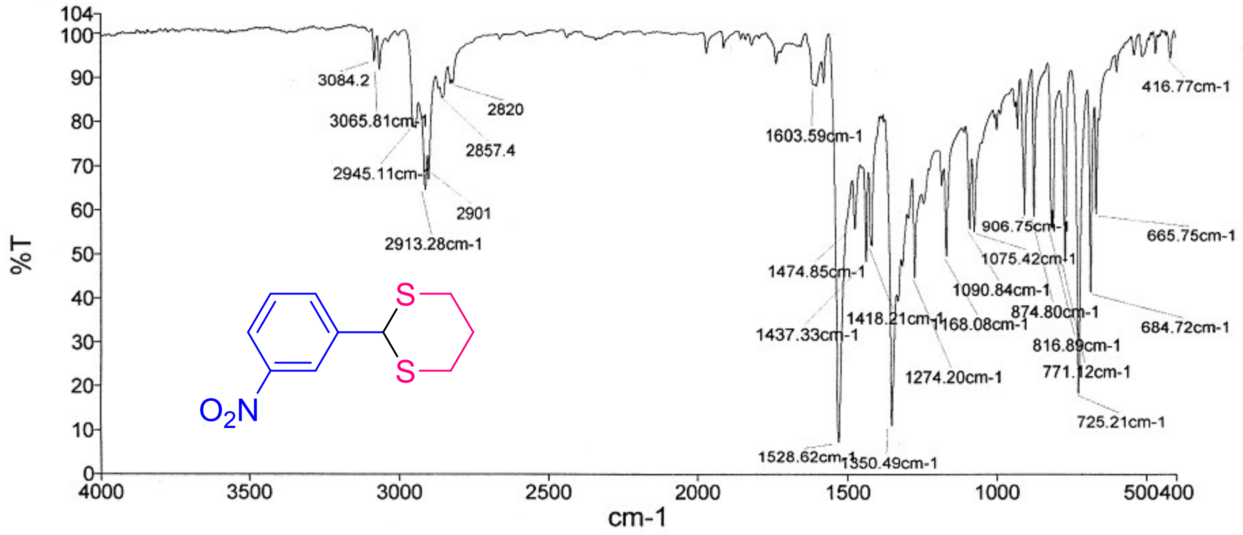

Figure 49. FT-IR spectrum of 2-(3-nitrophenyl)-1,3-dithiane (2b)

2-(4-(trifluoromethyl)phenyl)-1,3-dithiane (2c)

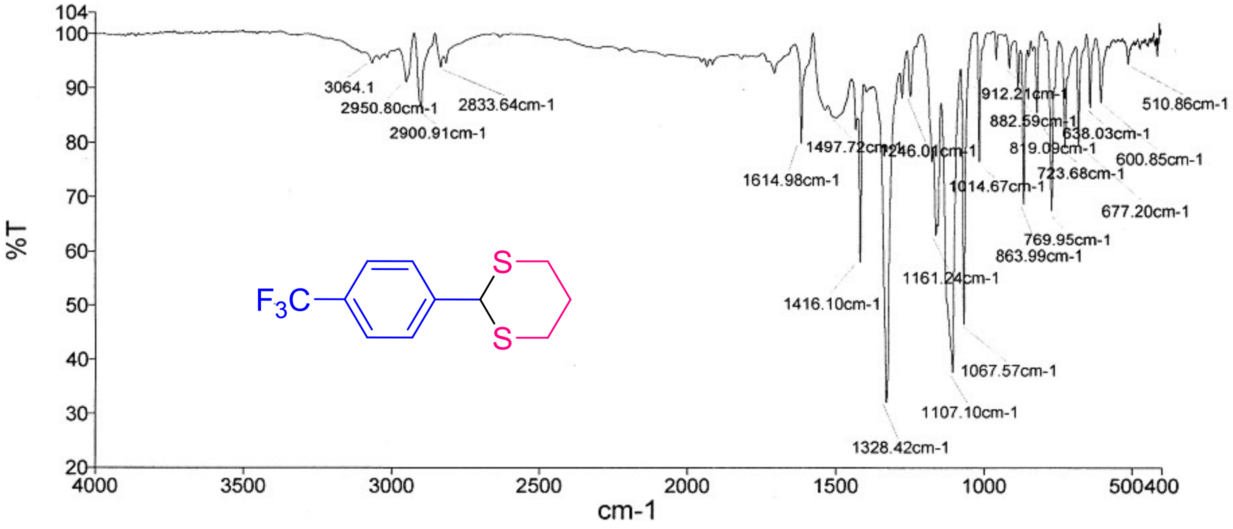

Figure 50. FT-IR spectrum of 2-(4-(trifluoromethyl)phenyl)-1,3-dithiane (2c)

2-(4-chlorophenyl)-1,3-dithiane (2d)

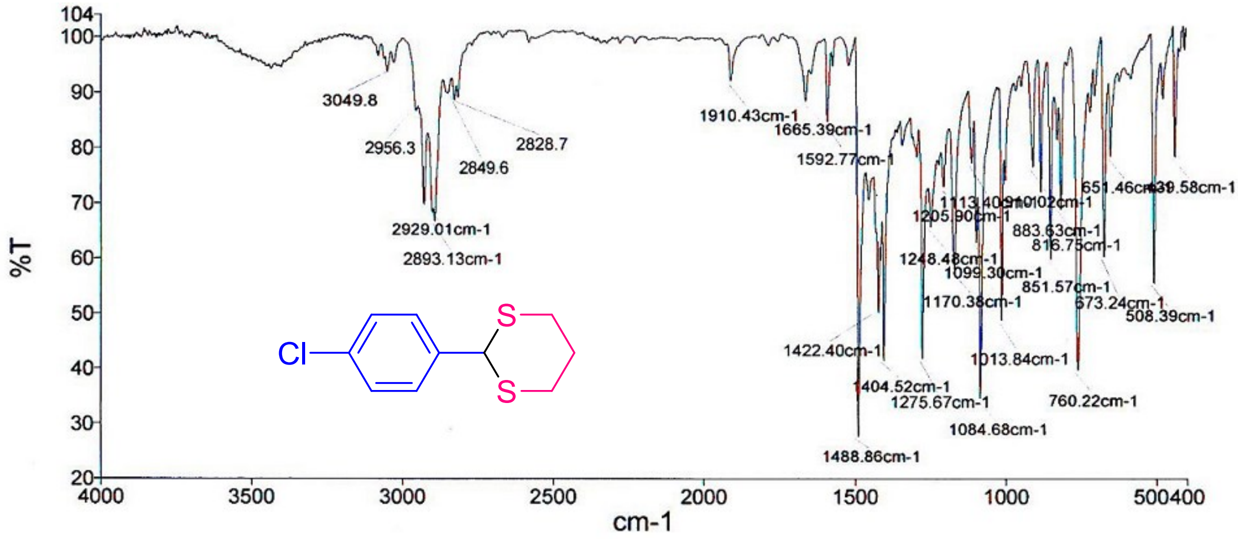

Figure 51. FT-IR spectrum of 2-(4-chlorophenyl)-1,3-dithiane (2d)

4-(1,3-dithian-2-yl)phenol (2e)

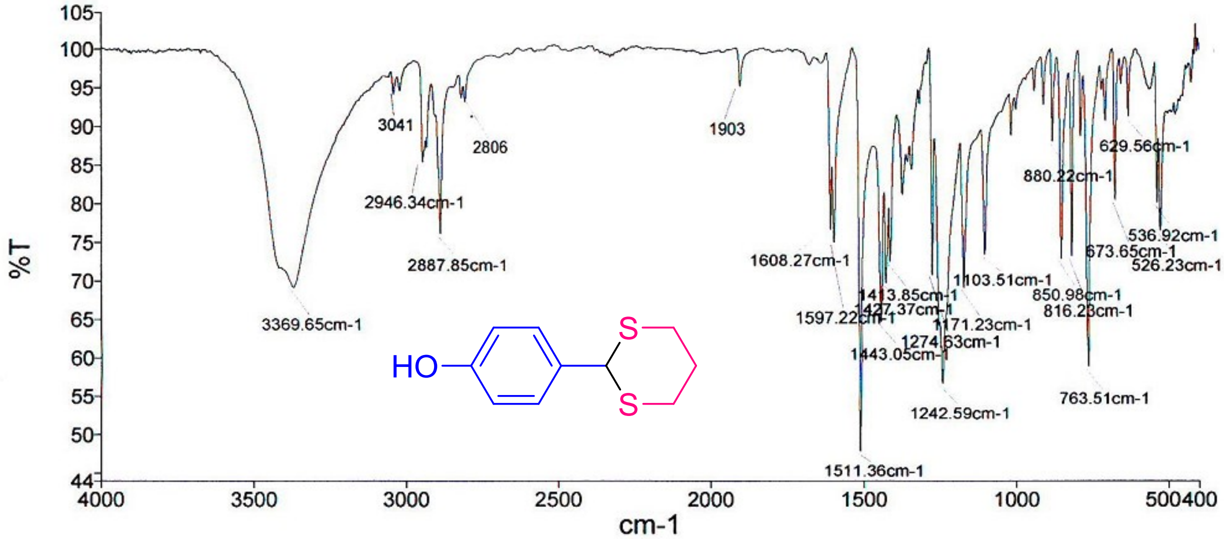

Figure 52. FT-IR spectrum of 4-(1,3-dithian-2-yl)phenol (2e)

2-(p-tolyl)-1,3-dithiane (2f)

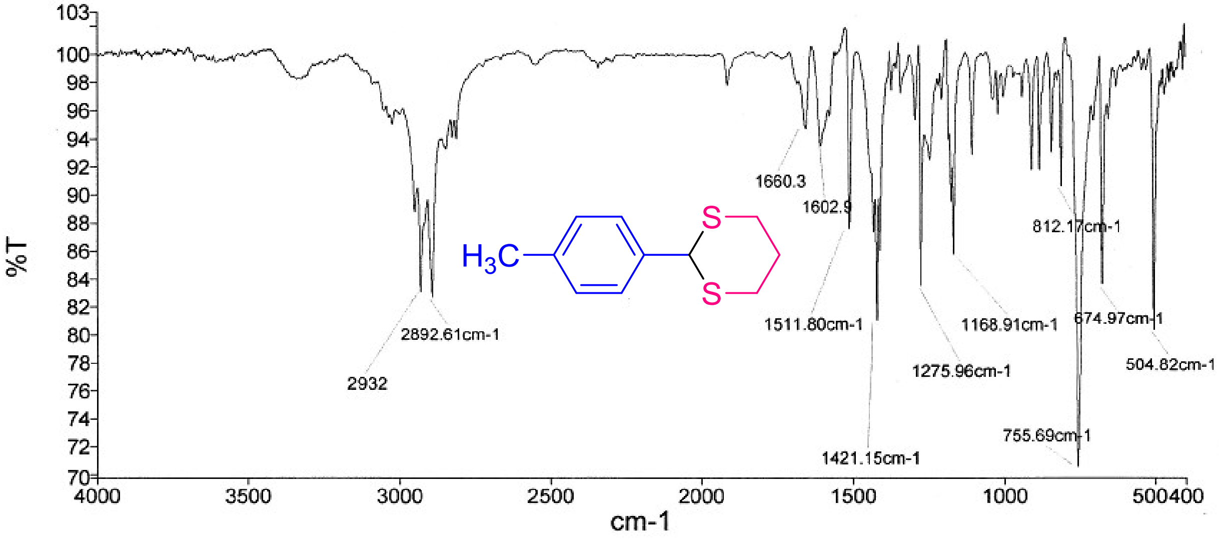

Figure 53. FT-IR spectrum of 2-(p-tolyl)-1,3-dithiane (2f)

2-phenyl-1,3-dithiane (2g)

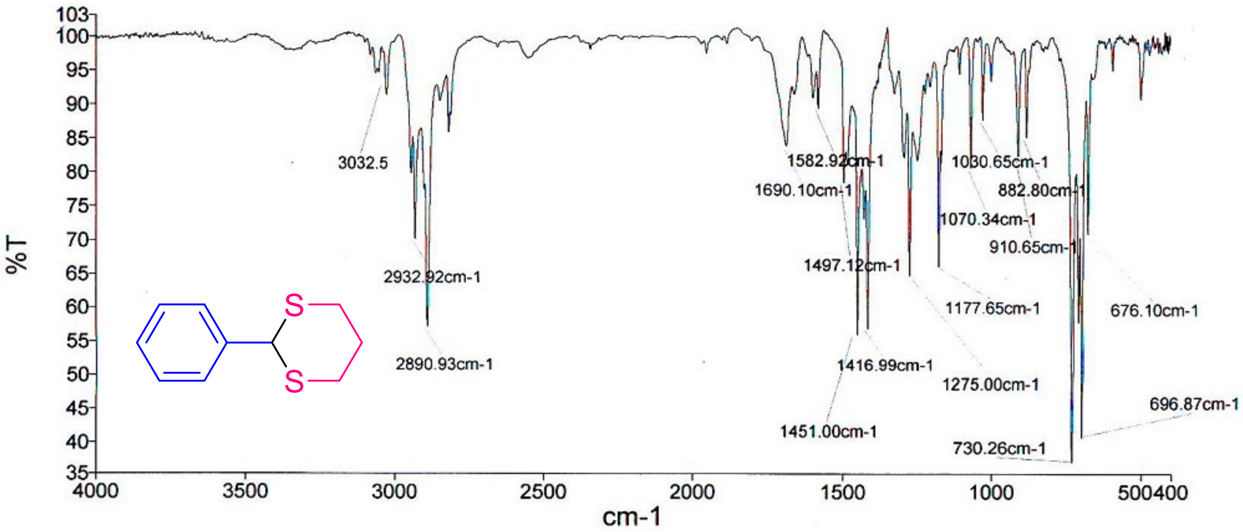

Figure 54. FT-IR spectrum of 2-phenyl-1,3-dithiane (2g)

2-(naphthalen-2-yl)-1,3-dithiane (2h)

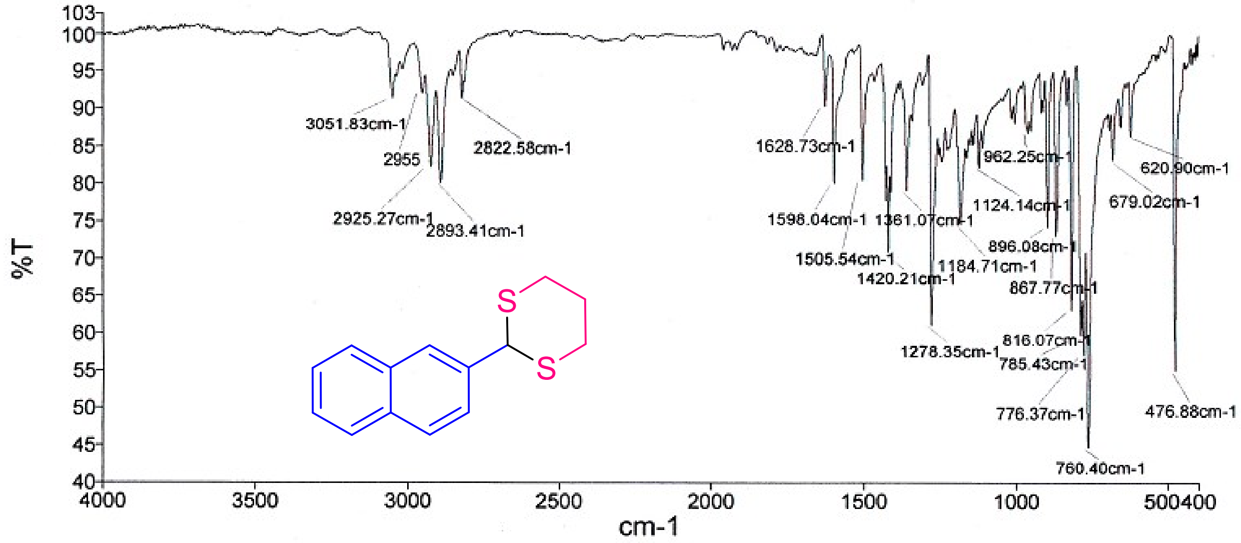

Figure 55. FT-IR spectrum of 2-(naphthalen-2-yl)-1,3-dithiane (2h)

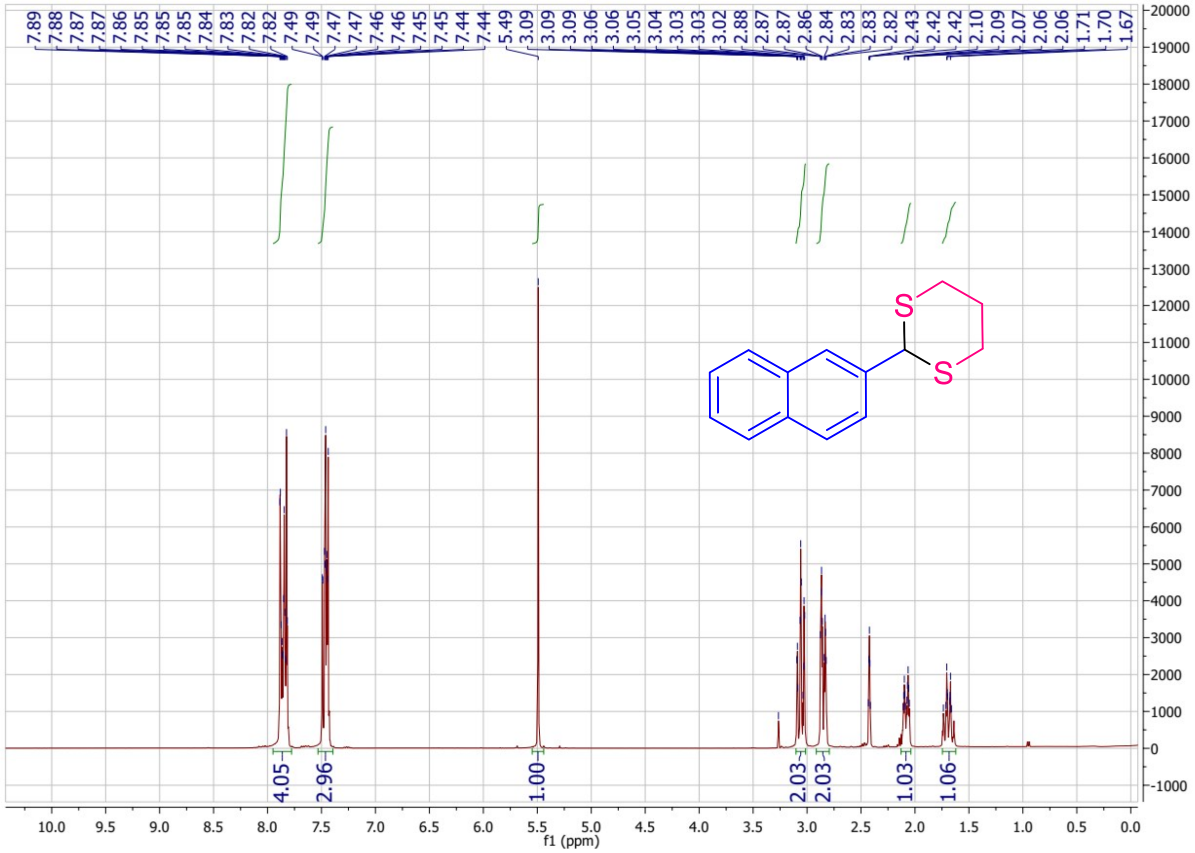

Figure 56. ^{1}H NMR spectrum of 2-(naphthalen-2-yl)-1,3-dithiane (2h)

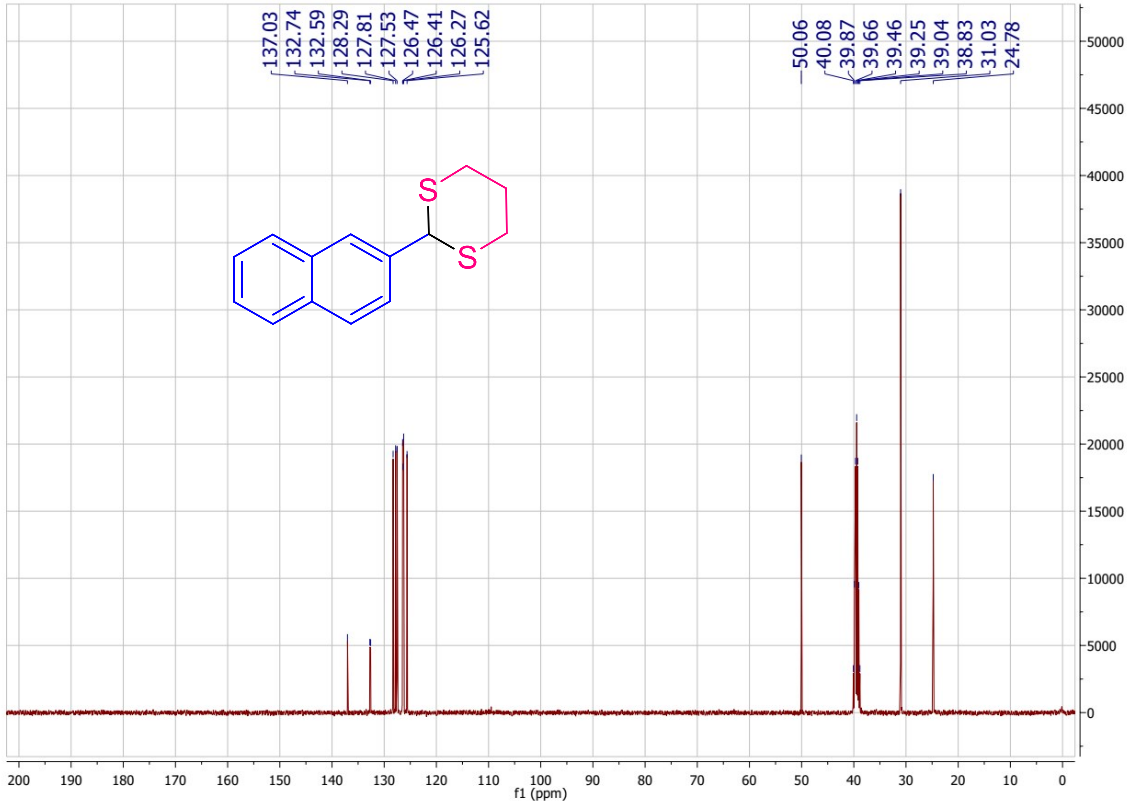

Figure 57. ^{13}C NMR spectrum of 2-(naphthalen-2-yl)-1,3-dithiane (2h)

2-methyl-2-(4-nitrophenyl)-1,3-dithiane (2i)

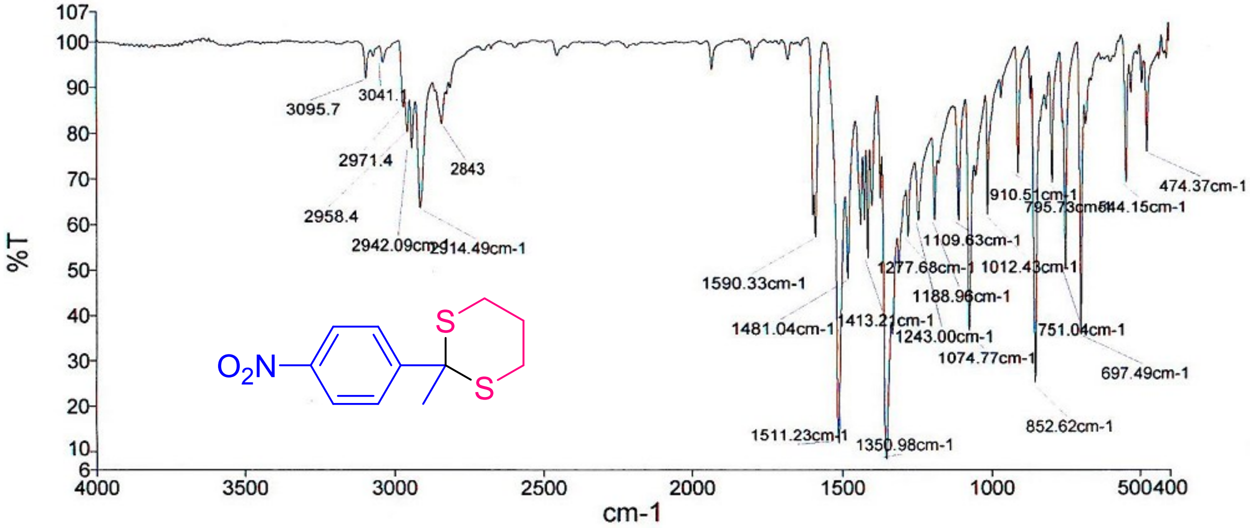

Figure 58. FT-IR spectrum of 2-methyl-2-(4-nitrophenyl)-1,3-dithiane (2i)

((methoxymethoxy)methyl)benzene (3a)

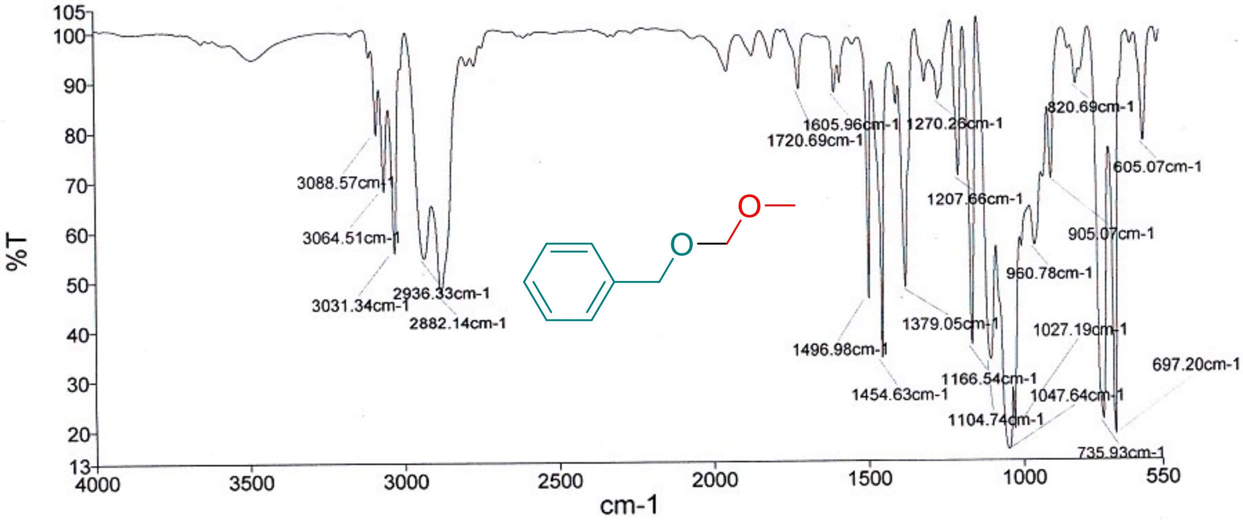

Figure 59. FT-IR spectrum of ((methoxymethoxy)methyl)benzene (3a)

1-methoxy-4-((methoxymethoxy)methyl)benzene (3b)

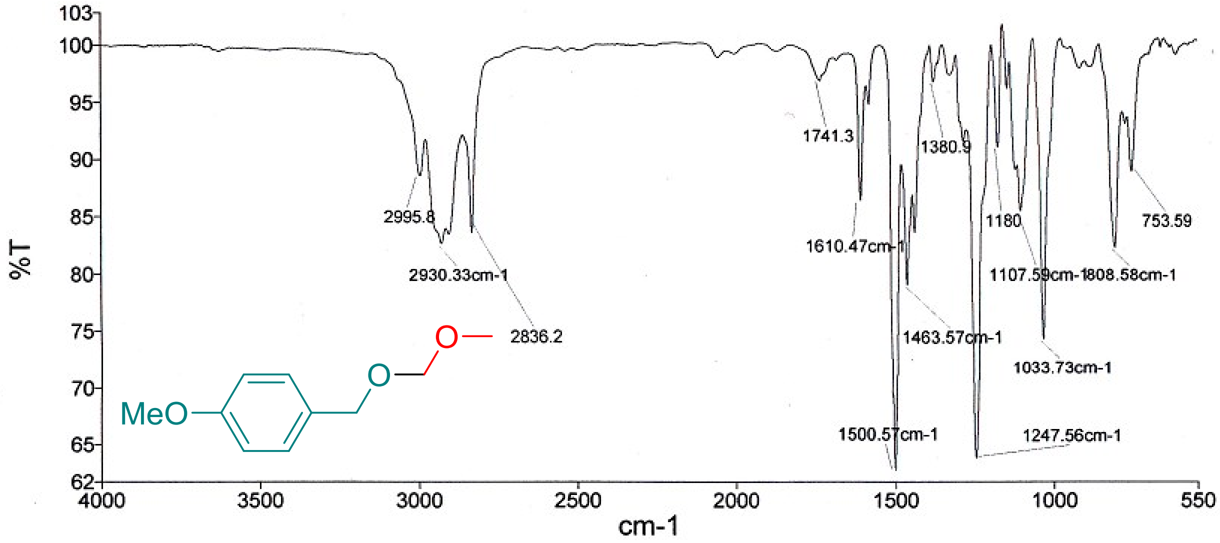

Figure 60. FT-IR spectrum of 1-methoxy-4-((methoxymethoxy)methyl)benzene (3b)

1-chloro-4-((methoxymethoxy)methyl)benzene (3c)

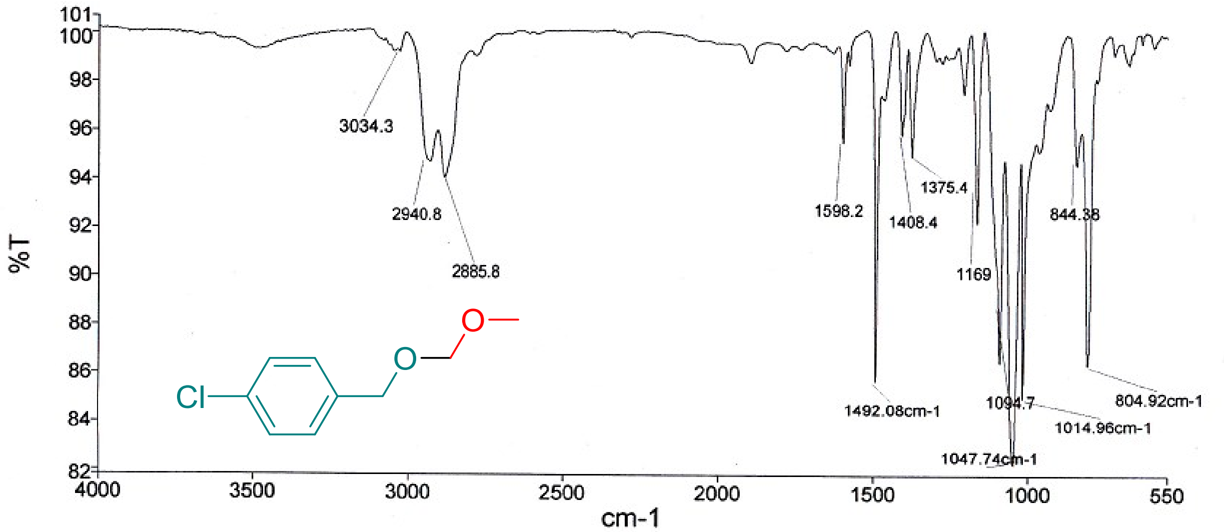

Figure 61. FT-IR spectrum of 1-chloro-4-((methoxymethoxy)methyl)benzene (3c)

4-((methoxymethoxy)methyl)phenol (3d)

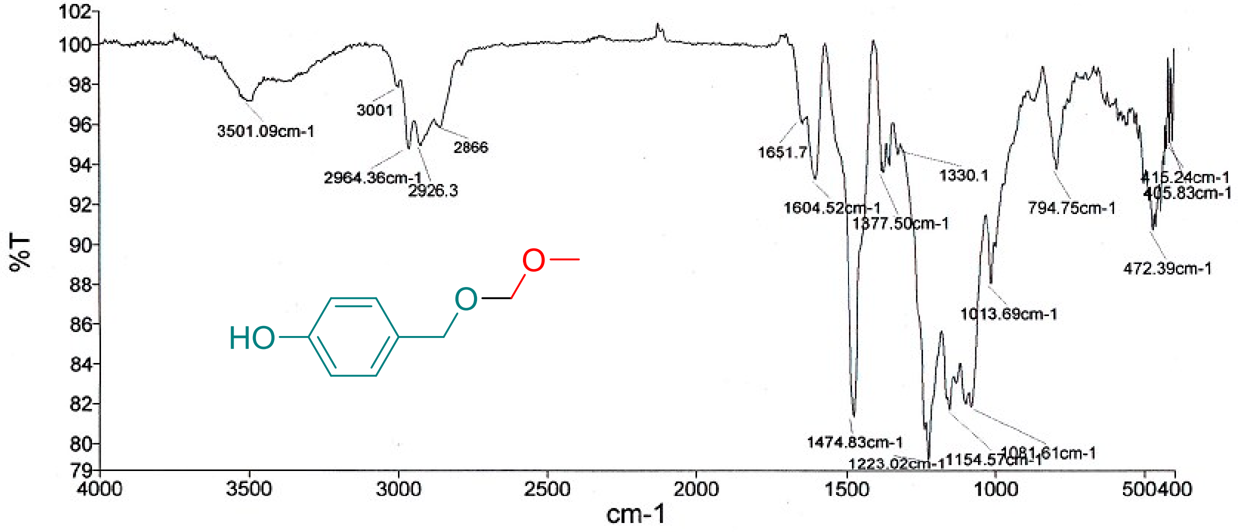

Figure 62. FT-IR spectrum of 4-((methoxymethoxy)methyl)phenol (3d)

1-bromo-2-((methoxymethoxy)methyl)benzene (3e)

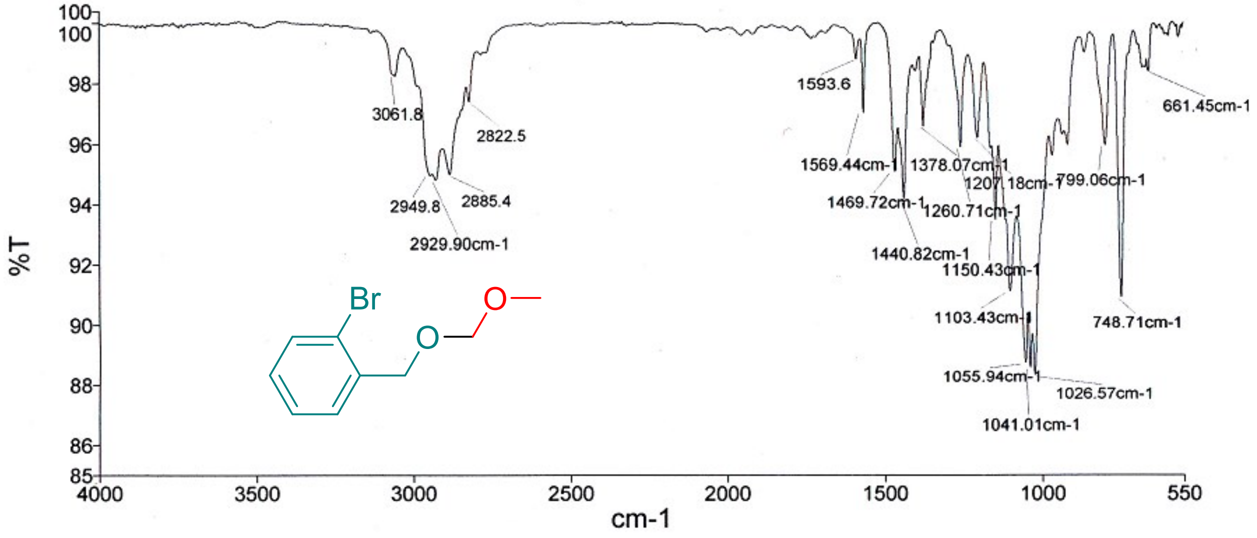

Figure 63. FT-IR spectrum of 1-bromo-2-((methoxymethoxy)methyl)benzene (3e)

1-((methoxymethoxy)methyl)-2-methylbenzene (3f)

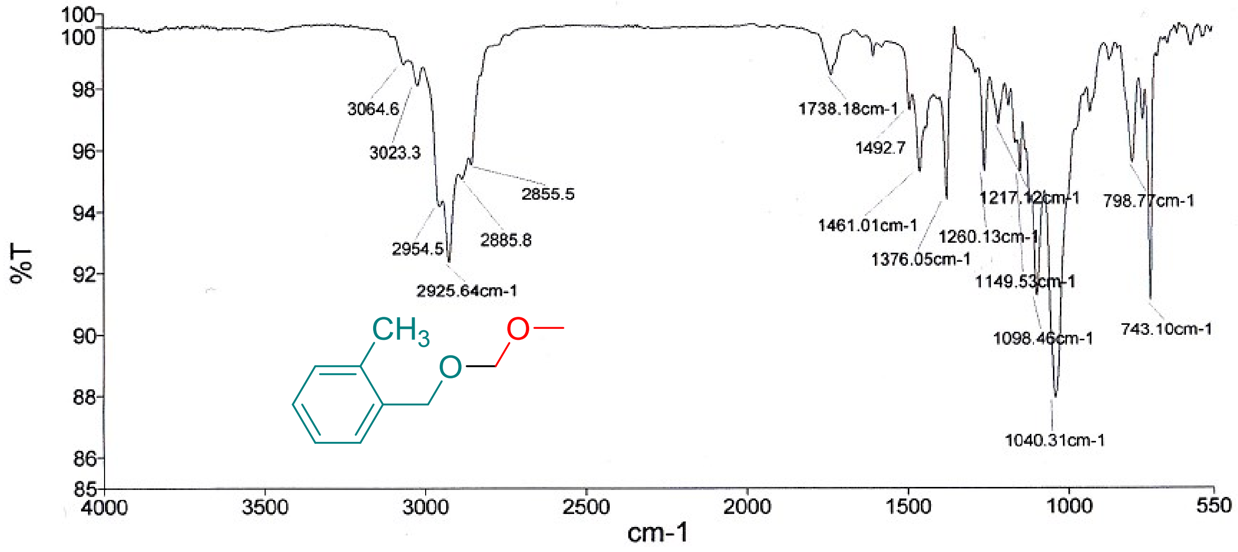

Figure 64. FT-IR spectrum of 1-((methoxymethoxy)methyl)-2-methylbenzene (3f)

2-((methoxymethoxy)methyl)phenol (3g)

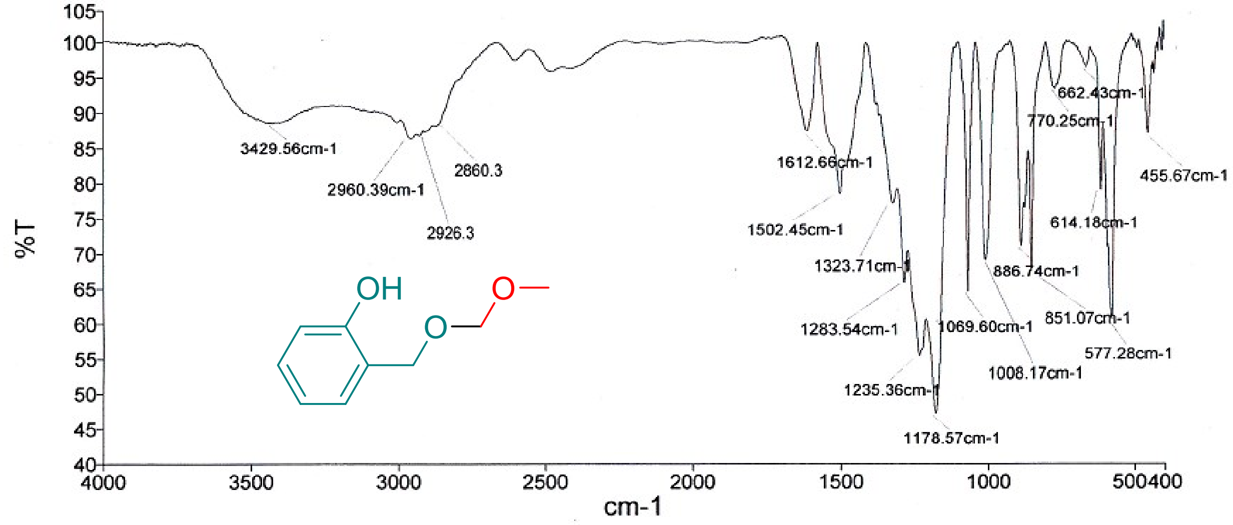

Figure 65. FT-IR spectrum of 2-((methoxymethoxy)methyl)phenol (3g)

1-(methoxymethoxy)butane (3h)

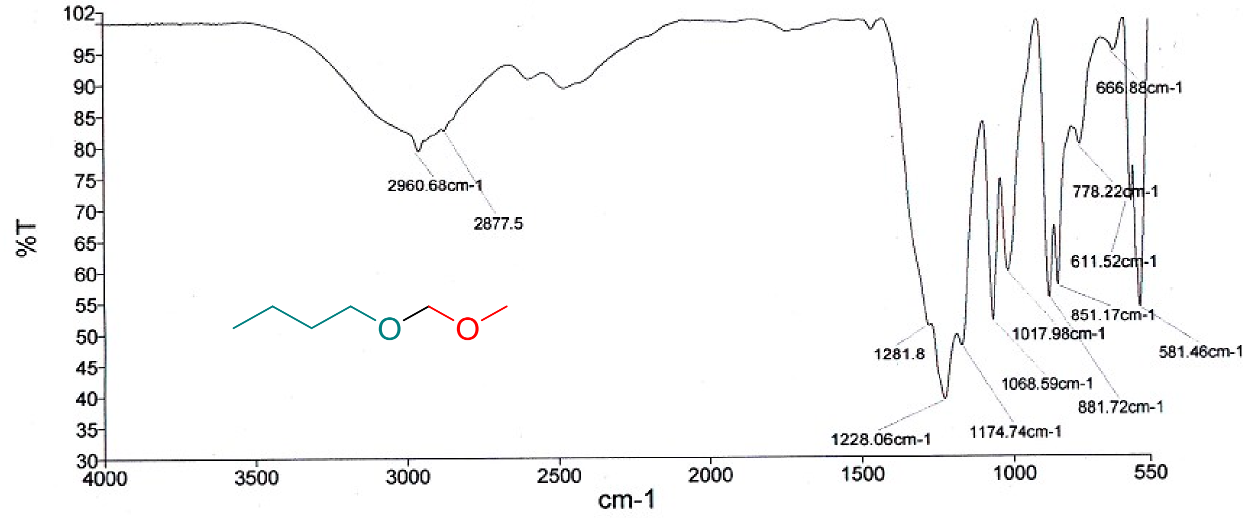

Figure 66. FT-IR spectrum of 1-(methoxymethoxy)butane (3h)
